# Development and validation of a CRISPR interference system for gene regulation in *Campylobacter jejuni*

**DOI:** 10.1186/s12866-022-02645-4

**Published:** 2022-10-05

**Authors:** Ruby Costigan, Emily Stoakes, R. Andres Floto, Julian Parkhill, Andrew J. Grant

**Affiliations:** 1 Department of Veterinary Medicine, University of Cambridge, Cambridge, UK; 2 Department of Medicine, MRC-Laboratory of Molecular Biology, Molecular Immunity Unit, University of Cambridge, Cambridge, UK; 3 University of Cambridge, Centre for AI in Medicine, Cambridge, UK; 4 Cambridge Centre for Lung Infection, Papworth Hospital, Cambridge, UK

**Keywords:** *Campylobacter jejuni*, CRISPRi, gene regulation

## Abstract

**Background:**

*Campylobacter* spp. are the leading cause of bacterial food-borne illness in humans worldwide, with *Campylobacter jejuni* responsible for 80% of these infections. There is an urgent need to understand fundamental *C. jejuni* biology for the development of new strategies to prevent and treat infections. The range of molecular tools available to regulate gene expression in *C. jejuni* is limited, which in turn constrains our ability to interrogate the function of essential and conditionally essential genes. We have addressed this by developing and utilising a CRISPR-based interference system known as CRISPRi in *C. jejuni* to control gene expression. To achieve this, a catalytically inactive (“dead”) *cas9* and sgRNA backbone from the *Streptococcus pyogenes* CRISPRi system was combined with *C. jejuni-*derived promoters of predetermined expression activities to develop a CRISPRi-based repression tool in *C. jejuni* strains M1Cam and 81–176.

**Results:**

The CRISPRi tool was validated through successful repression of the arylsulphatase-encoding gene astA using a range of sgRNA target sequences spanning the astA gene. The tool was also applied to target *astA* in an M1Cam CRISPR-Cas9 deletion strain, which showed that the presence of an endogenous CRISPR-Cas9 system did not affect the activity of the CRISPRi-based repression tool. The tool was further validated against the hippicurase-encoding gene *hipO*. Following this, the flagella genes *flgR*, *flaA*, *flaB* and both *flaA* and *flaB* were targeted for CRISPRi-based repression, which resulted in varying levels of motility reduction and flagella phenotypes as determined by phenotypical assays and transmission electron microscopy (TEM).

**Conclusions:**

This is the first report of a CRISPRi-based tool in *C. jejuni*, which will provide a valuable resource to the *Campylobacter* community.

**Supplementary Information:**

The online version contains supplementary material available at 10.1186/s12866-022-02645-4.

## Background

*Campylobacter* spp. infection is the leading cause of bacterial food-borne illness in humans worldwide[1], with *Campylobacter jejuni* responsible for ~ 80% of infections[2, 3]. Campylobacteriosis presents as acute gastroenteritis which, although typically self-limiting, can warrant antibiotic treatment. Moreover, accumulating evidence associates campylobacteriosis with chronic sequelae including Guillain-Barré syndrome[4, 5] and inflammatory bowel disease[6, 7]. Consequently, there is an urgent requirement to understand fundamental *C. jejuni* biology and transmission routes for the development of new strategies to prevent and treat infections.

The implementation of transposon (Tn) mutagenesis in *C. jejuni* has provided key insights into genes essential for growth and survival[8]. It has also identified several essential or conditionally essential genes of unknown function. There are limited molecular tools available to regulate gene expression in *C. jejuni*, which in turn hinders investigation into the function of essential and conditionally essential genes. As deletion or inactivation of these genes will likely result in a lethal phenotype, the means to regulate the expression of target genes would provide a useful step forward in gene function analysis in *C. jejuni*. We aimed to address this by establishing and validating a CRISPR interference (CRISPRi)-based repression tool in *C. jejuni* to control gene expression.

CRISPRi utilises a catalytically inactive ("dead”) Cas9 protein (dCas9) along with an sgRNA to provide repression of target genes. Whilst conventional CRISPR-Cas9 technology performs targeted double-stranded cleavage of DNA, CRISPRi uses a sgRNA-dCas9 complex which binds to the target sequence and prevents transcription via steric hindrance of the RNA polymerase protein complex[9]. CRISPRi has been successfully developed in a wide range of Gram-negative and Gram-positive bacteria[10], including *Escherichia coli*[9, 11–13]*, Bacillus subtilis*[14, 15])*, Staphylococcus aureus*[16–18]*, Mycobacteria* spp[19–22] and *Vibrio cholerae*[23].

A CRISPRi construct constitutively expressing both dCas9 and sgRNA was designed, informed by an activity analysis of candidate *C. jejuni* promoters, and integrated into the chromosome of the *C. jejuni* strains M1Cam[24, 25] and 81–176[26]. The *S. pyogenes-*derived CRISPRi tool was validated against the target gene *astA*, which encodes an arylsulphatase enzyme that releases sulphates from aryl compounds. Importantly, its activity can be detected in *C. jejuni* through growth on the chromogenic substrate 5‐bromo‐4‐chloro‐3‐indolyl sulphate (XS)[27], and can be quantified using an nitrophenol assay, which measures the cleavage of nitrophenol from potassium 4-nitrophenyl sulfate by AstA in the presence of tyramine[27, 28]. These phenotypic readouts provided a convenient visual assessment of CRISPRi system functioning (XS agar) alongside a quantitative assessment of AstA activity as an indicator of *astA* transcription (nitrophenol assay) when assessing *C. jejuni* CRISPRi strains.

CRISPRi-based repression was developed in two *C. jejuni* strains, 81–176 and M1Cam. It is important to note that whilst M1Cam contains an endogenous Type II-C CRISPR-Cas9 system, 81–176 does not[29]. Therefore, the use of these two strains not only permitted demonstration of cross-strain functionality of the system, but also enabled the opportunity to explore whether the presence of a native CRISPR-Cas9 could impact functionality of exogenous CRISPRi machinery. This was achieved through the generation of a CRISPR-Cas9 locus deletion in M1Cam.

A key component of the validation experiments was to consider the suitable expression levels of both dCas9 and sgRNAs. It has been shown that excessive expression of *S. pyogenes* dCas9 in other bacterial species can be toxic[30]. However, sufficient expression of dCas9, as well as sgRNAs, must be achieved in order to create a system that delivers adequate levels of repression[9, 30]. Therefore, an analysis of candidate *C. jejuni* promoters for use in a CRISPRi system was performed using a LacZ reporter system. This enabled the selection of appropriate promoters to express *dcas9* and sgRNA within the CRISPRi constructs. Following this, target regions in the *astA* gene were identified and used to design a set of sgRNAs. The sgRNAs and *dcas9* were used to construct a series of homologous recombination plasmids, designed to deliver the CRISPRi machinery into a pseudogenic region (CjM1_0055-0056/57) in the chromosome of 81–176 and M1Cam. Following this, the *astA* phenotype of the resulting CRISPRi strains was qualitatively and quantitively evaluated using chromogenic agar plating and nitrophenol assays respectively. Finally, the impact of the endogenous CRISPR-Cas9 system in M1Cam was investigated, through deletion of *cas2, cas1, cas9* and the CRISPR array. A series of CRISPRi strains targeting *astA* were generated in the deletion strain to allow comparison of AstA activity in the presence and absence of the endogenous CRISPR-Cas9 system.

Following *astA* targeting in M1Cam and 81–176, the CRISPRi-based repression tool was further validated through targeting of the gene *hipO*, which encodes a hippicurase enzyme. Hippicurase is an enzyme which converts hippuric acid into benzoic acid and glycine[31] and is present in both M1Cam and 81–176. Detection of its activity can be achieved through a hippicurase assay[32]. During the assay, bacterial cells are lysed to release the hippicurase enzyme. Following this, HipO activity is assessed through the addition of ninhydrin, which reacts with glycine and results in the formation of a purple product, which is measured using a spectrophotometer at 570 nm. A series of CRISPRi strains in M1Cam and 81–176 were generated to target *hipO* at a range of locations from 5’ to 3’, and the hippicurase assay was used to assess HipO activity in each.

Next, genes from the flagella biosynthetic pathway were selected for CRISPRi-based repression. Flagella biosynthesis is a tightly regulated process, whereby following formation of the flagellar T3SS core, the FlgSR response-regulator system is activated through FlgS auto-phosphorylation and subsequent FlgR phosphorylation. FlgR then triggers the expression of σ^54^ Class II genes, although the mechanism by which it performs this is unclear [33, 34]. Expression of σ^54^ Class II genes results in the production of the minor flagella filament protein FlaB, and the remaining hook basal body components including the rod and the hook. Following this, the regulator protein FlgM is secreted, lifting its repression of σ^28^, which then mediates expression of Class III genes, including the major flagella filament gene *flaA*, resulting in the assembly of the complete flagellum[35].

This pathway was targeted for several reasons. Firstly, motility is a key determinant of virulence in *C. jejuni*[36, 37], and therefore the flagella biosynthesis pathway is a heavily researched area within the field. Despite this, gaps still exist in the understanding of the regulation of flagella biosynthesis[38, 39]. If CRISPRi-based repression of genes involved in this pathway could be achieved, this would offer a useful tool to explore regulation of flagellar assembly in greater depth. Secondly, from a practical level, *C. jejuni* motility can be measured using soft-agar stab motility assays, which therefore provided a phenotypic screen to assess the levels of CRISPRi-based repression of genes within the flagella biosynthesis pathway. Finally, although the CRISPRi-based repression tool has been verified through targeting of *hipO* and *astA*, applying it to genes within the flagella biosynthesis pathway provided the opportunity to validate the tool with a more complex regulatory system which has still not been fully resolved.

Within the flagella biosynthesis pathway, the genes *flgR*, *flaA* and *flaB* were selected for CRISPRi-based repression. The rationale for choosing these genes was as follows: Firstly, *flgR* was chosen as its deletion has previously been shown to abolish motility[40], meaning that complete repression of *flgR* was predicted to result in the same phenotype. In turn, incomplete repression was expected to result in a reduction in motility. Secondly, *flaA* and *flaB* were selected as their products form the external flagellar filament[41, 42], and their expression lies further down the flagella biosynthetic pathway in comparison to *flgR*[33], meaning that the pathway could be targeted at multiple points.

As well as being targeted for CRISPRi-based repression individually, *flaA* and *flaB* were also targeted simultaneously using a single sgRNA target location, as the genes share 92.5% sequence homology in both M1Cam and 81–176. The motility of all CRISPRi transformants was measured using soft-agar motility plating to assess the impact of CRISPRi-based repression on the flagella biosynthesis pathway. Following this, selected CRISPRi transformants were further analysed using RT-qPCR to gain insights into the expression levels of target genes, and transmission electron microscopy (TEM) was used to observe the phenotype of selected populations of bacteria.

In summary, we have developed and validated a novel CRISPRi tool for targeted transcriptional repression in *C. jejuni.*

## Results

### Activity screening of candidate *C. jejuni* promoters using a LacZ reporter system

**Fig. 1:**
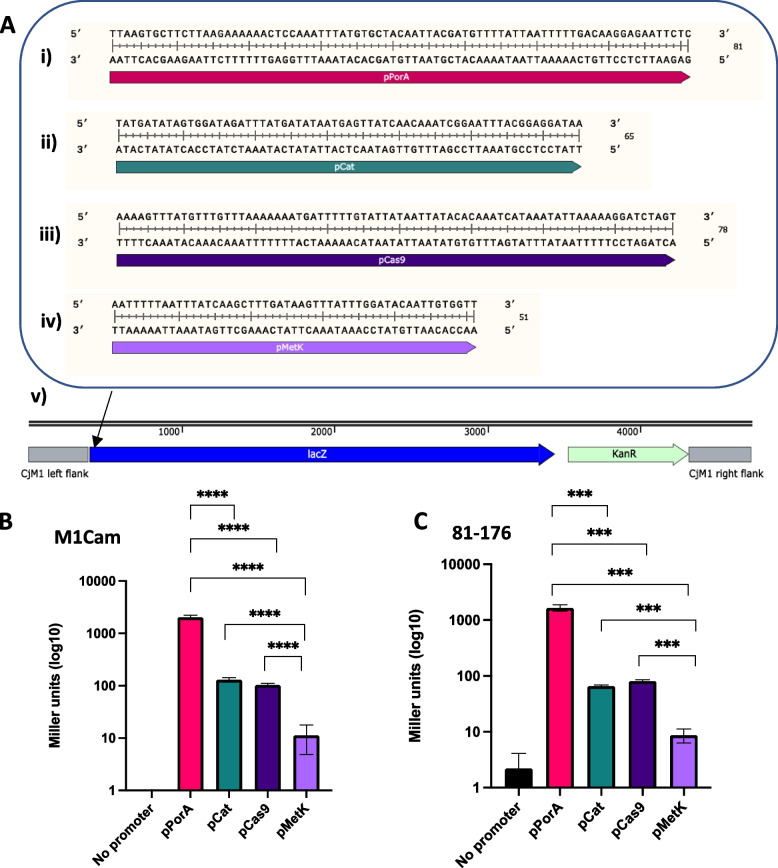
Activity screening of candidate C. jejuni promoters in M1Cam and 81–176 using a LacZ reporter system*.* Four *C. jejuni* promoters, pPorA (A,i), pCatA (A,ii), pCas9 (A,iii) and pMetK (A,iv) were each inserted into a *lacZ* Kan^r^ reporter construct (v). The reporter constructs were transformed into M1Cam and 81–176 and screened using a β-galactosidase assay (B and C, respectively). In both strains, pPorA demonstrated the greatest activity, followed by pCat and pCas9 which displayed similar activities, and pMetK, which had the lowest activity. **** = *p* < 0.0001, *** = *p* < 0.001. *N* = 3 biological replicates, Statistics performed using an unpaired t-test, error bars = SD

To identify promoters for appropriate expression levels of *S. pyogenes dcas9* and sgRNA, a series of candidate *C. jejuni* promoters were assessed for their expression activities using a LacZ reporter assay (Fig. 1A, i-iv). The promoters chosen for this analysis included those driving the following genes; *porA* (major outer membrane protein), *cat* (chloramphenicol acetyltransferase resistance cassette), *cas9* (CRISPR-associated protein 9) and *metK* (methionine adenosyltransferase). These promoters are hereafter referred to as pPorA, pCat, pCas9 and pMetK. The promoters pPorA and pCat were selected due to our previous experience[25], with pPorA being considered a “high-strength” promoter and pCat a “low-strength” promoter. pCas9 was chosen in order to gain an insight into the endogenous levels of expression of *C. jejuni cas9*. Finally, pMetK was chosen due to its previous use to express *cas9* in complementation strains[43].

To enable comparative assessment of activity, each promoter was cloned with a *lacZ* gene and transformed into a pseudogenic chromosomal region in 81–176 and M1Cam using the homologous recombination vector pRC1 (Fig. 1A). A no promoter control was included to detect any promotor read-through that could contribute to *lacZ* expression in the pseudogenic site. Together, this resulted in a panel of 5 transformants per *C. jejuni* strain which were then assessed for LacZ activity using a β-galactosidase assay protocol previously developed in *C. jejuni* to monitor plasmid-based LacZ expression[44].

As expected, the results of the β-galactosidase assay (Fig. 1B &C) showed that in both strains, pPorA had the greatest activity, with a significantly higher activity than any of the other promoters, in both M1Cam and 81–176. pCat and pCas9 both showed a similar level of activity in both strains, demonstrating “moderate-strength” activities within the range of the promoter panel tested. Finally, pMetK displayed the lowest activity amongst the promoter panel, with a significantly weaker level of LacZ activity in comparison to pPorA, pCat and pCas9 in both M1Cam and 81–176. Interestingly, a small amount of LacZ activity was detected in the “no promoter” 81–176 strain, indicating that there may be some readthrough from promoters upstream of this region. Overall, the results of the assay provided quantitative assessment of activity of four *C. jejuni* promoters in strains 81–176 and M1Cam.

### Assembly of CRISPRi components into constructs for transformation into *C. jejuni*

From the data gathered on promoter activities using the β-galactosidase assay, the promoters pCat and pPorA were chosen to express *dcas9* and sgRNA, respectively. pCat was chosen to express *dcas9* as firstly, it showed a similar level of expression in comparison to the endogenous *C. jejuni cas9* promoter, and secondly, it demonstrated a moderate level of expression in comparison to pPorA. This was preferable to avoid dCas9 overexpression whilst also allowing sufficient dCas9 production for repression to occur. pCat was chosen over pCas9 due to its extensive use in our previous studies. Meanwhile, pPorA was chosen to express the sgRNA component to saturate the system with target sgRNA and thereby prevent their levels being a limiting factor in repression.

**Fig. 2:**
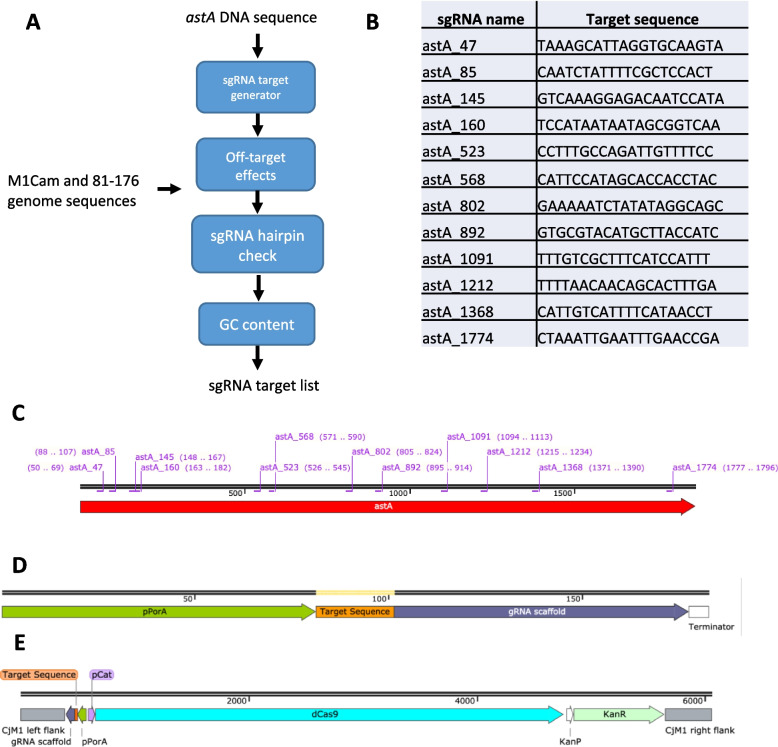
Design of sgRNA target sequences for integration into an sgRNA scaffold to combine with dcas9 to form a CRISPRi homologous recombination vector. An sgRNA target generator script was used to create a list of sgRNA targets using the *astA* gene sequence (A). Targets were screened for off-target effects using the genome sequences of 81–176 and M1Cam. Target sequences were then screened to detect any hairpin formation with the sgRNA scaffold backbone and selected for a GC content between 30–60%. The final list of 12 sgRNA target sequences are shown in (B), with their relative locations along the *astA* gene shown in (C). To express sgRNAs, a scaffold construct with a pPorA promoter and an sgRNA backbone was synthesized (D) and inserted into pRC1 in the opposite orientation to *dcas9* driven by pCat (E)

Next, the *astA* gene sequence was screened using a sgRNA target generator script[45] to identify a series of 20 bp sgRNA target sequences directly upstream of a 5’-NGG-3’ PAM site in the template strand of the gene, meaning the sgRNAs would target the non-template strand (Fig. 2A). The target generator script contained a series of parameters that were set to minimise the likelihood of off-target effects, which discarded any sgRNAs that were likely to disrupt the transcription of any genes other than *astA*. Following this, the output target sequences were passed through a sgRNA hairpin checker script[46] to detect hairpin formation between the target sequence and the backbone of the sgRNA. Target sequences where hairpin formation was detected were discarded. Finally, the GC content of the remaining target sequences were calculated and guides with a GC content below 30% and above 60% were discarded. This was because target sequences were integrated into CRISPRi plasmids as primers through inverse PCR, and therefore required an appropriate GC content to enable stable binding of primer and template. This culminated in a list of 14 sgRNA sequences shared by M1Cam and 81–176, targeting a series of regions from 5’ to 3’ along *astA*, each named astA_N, where N was the location of the first base of the PAM site adjacent to the target region. Although it has been suggested that targeting the 5’ end of genes of interest is the most effective strategy in CRISPRi sgRNA design[9], sgRNAs spanning the length of the gene were selected, as the system had not been trialled in *C. jejuni* before. However, 2 sgRNAs from the list of 14 were omitted; astA_1369 as it was only 1 bp downstream from astA_1368, and astA_1825 following multiple unsuccessful attempts to construct a plasmid containing this target sequence. This resulted in 12 sgRNA target sequences to be taken forward to generate CRISPRi constructs (Fig. 2B &C).

The *dcas9* and sgRNA scaffold components were assembled into the homologous recombination vector pRC1, facing in opposite orientations to prevent promoter read-through, to produce a CRISPRi construct constitutively expressing *dcas9* and an sgRNA (Fig. 2D &E). Subsequently, only the target region of the sgRNA scaffold was modified to produce each different CRISPRi construct, resulting in 12 plasmids – pRC40-44 and pRC73-80 (Table S2).

### Deletion of CRISPR-Cas9 locus in M1Cam

In preparation for the transformation of CRISPRi constructs into M1Cam, a deletion mutant of the CRISPR-Cas9 locus was generated to control for any competition between the endogenous CRISPR-Cas9 and the exogenous CRISPRi machinery. The CRISPR-Cas9 locus in M1Cam is a Type-II system and features *cas2*, *cas1* and *cas9* genes and a small CRISPR array[29]. A deletion vector, pRC_CRISPR (Table S2) containing ~ 500 bp left and right homologous flanking sequences to regions up- and down-stream to the 4737 bp CRISPR region and a chloramphenicol acetyltransferase resistance cassette (*cat*) were used to delete the region from the M1Cam genome. Constructs were confirmed and verified by whole genome sequence (WGS) analysis. This produced strain M1Cam ∆CRISPR_array/*cas2*/*cas1*/*cas9*::*cat*, hereafter referred to as M1Cam∆CRISPR. WGS showed successful deletion of the region but highlighted a single CTT deletion in an intergenic region located at 1,279,352 bp. The intergenic region was between *CjM1Cam_1315*, encoding a putative plasmid stabilisation system protein and *CjM1Cam_1315*, a tRNA-Ser, which is encoded on the reverse strand, meaning that the CTT deletion was downstream of each coding region. SNPs and INDELs can appear during growth and passage of *C. jejuni*[25, 47, 48], therefore M1Cam∆CRISPR was taken forward for the generation of CRISPRi transformants.

### Assessment of CRISPRi-based repression of *astA* using a nitrophenol assay

Constructs pRC40-44 and pRC73-80 (Table S2) were transformed into a pseudogenic chromosomal region in 81–176, M1Cam and M1Cam∆CRISPR (CjM1_0055-56/0057) to produce 12 CRISPRi transformants targeting *astA* per strain. Each transformant was named after the sgRNA within the CRISPRi plasmid it had been transformed with (*e.g.* astA_47) (Table S1). Plating of the transformants onto MH agar supplemented with 100 µg/ml XS enabled qualitative assessment of AstA activity in each strain, with a reduced level of blue colouration of the bacterial growth to varying degrees seen in all 12 CRISPRi transformants across all 3 strains (Fig S1).

**Fig. 3:**
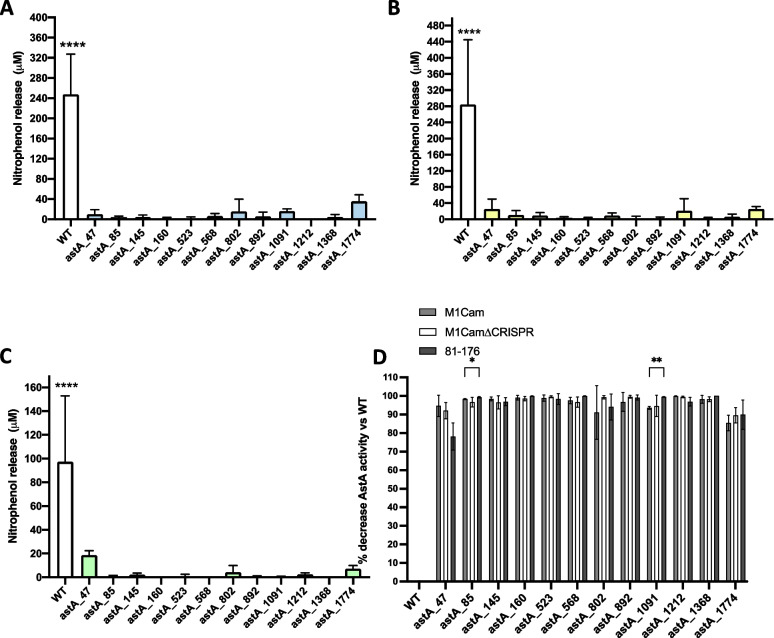
Quantification and comparison of AstA activity in 81–176, M1Cam and M1Cam∆CRISPR CRISPRi transformants using a nitrophenol assay. A significant decrease in AstA activity was demonstrated for each sgRNA in all CRISPRi transformants (A, B and C) in comparison to each respective WT (**** = *P* < 0.0001, ANOVA, *N* = 3, error bars = SD). Mean percentage decrease calculations for each CRISPRi transformant with respect to the WT in each strain showed no significant difference in CRISPRi efficacy between M1Cam and M1Cam∆CRISPR (D). All sgRNA target locations showed > 78% decrease in detectable AstA activity across all strains. No significant difference in sgRNA efficacy was detected between strains except for astA_85 and astA_1091 between M1Cam and 81–176 (* = *p* < 0.05, 2-way ANOVA, error bars = SD)

To quantify this observation, nitrophenol assays were performed for each set of transformants. For M1Cam, M1Cam∆CRISPR and 81–176, the nitrophenol assay results demonstrated a significant decrease in AstA activity for all sgRNAs compared to their respective parental wild-type (WT) (Fig. 3 A, B & C). Although the efficacy of *astA* repression appeared to differ between sgRNA target location, this did not prove to be statistically significant. Interestingly, the overall levels of AstA activity within M1Cam were greater than 81–176. This could be seen visually during culture on chromogenic XS agar (Fig S1) and upon quantification with nitrophenol assays, with a mean nitrophenol release of 247.6 µM, 284.6 µM and 97.3 µM for M1Cam, M1Cam∆CRISPR and 81–176, respectively (Fig. 3A, B & C).

Next, to assess if there was any potential competition between the endogenous CRISPR machinery in M1Cam and the exogenous CRISPRi components that affected CRISPRi repression efficiency, AstA activity levels between M1Cam and M1Cam∆CRISPR strains were compared. The efficacy of each sgRNA for each strain was compared by calculating the mean percentage decrease in AstA activity with respect to each parental WT strain. The percentage decrease for each sgRNA was then compared between strains (Fig. 3D). There was no statistically significant difference between M1Cam and M1∆CRISPR AstA activity decreases for each sgRNA. Implying that there was not competition between the endogenous CRISPR-Cas9 and the exogenous CRISPRi machinery.

Comparisons between the M1Cam, M1Cam∆CRISPR strains and 81–176 strains were performed to assess the efficacy of each sgRNA location between strains. This showed that there was no significant difference in sgRNA efficacy except for sgRNA astA_85 and sgRNA_1091 between M1Cam and 81–176, whereby 81–176 had a greater decrease in AstA activity than M1Cam with these sgRNAs. Additionally, the analysis showed that all sgRNAs provided at least 78% decrease in activity across all strains (Fig. 3D), highlighting the repression efficacy of the CRISPRi-based repression system.

### Further validation of CRISPRi-based repression targeting the hippicurase gene *hipO*

**Fig. 4:**
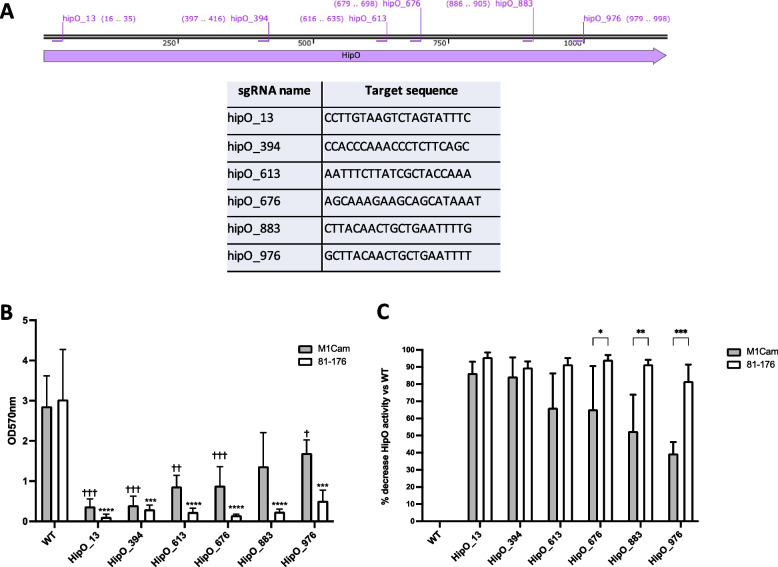
Further validation of CRISPRi-based repression tool through targeting of *hipO*. Six target sgRNA locations were identified in the *hipO* gene in M1Cam and 81–176, distributed from the 5’ to 3’ end of the gene (A). Assessment of HipO activity in M1Cam and 81–176 CRISPRi strains targeting *hipO* in a range of locations (B). All target locations in 81–176 CRISPRi strains showed significantly decreased HipO activity (**** = *p* < 0.0001, *** = *p* < 0.001, one way ANOVA). All target locations except HipO_883 showed significantly decreased HipO activity in M1Cam CRISPRi strains (††† = *p* < 0.001, †† = *p* < 0.01, † = *p* < 0.05, one way ANOVA), with a trend of decreased activity from 5’ to 3’, although no significant difference was found between sgRNA target locations. 81–176 – *N* = 3 biological replicates, M1Cam – *N* = 5 biological replicates, error bars = SD. Analysis of relative percentage decrease in HipO activity for each strain (C) revealed that the sgRNAs HipO_676, HipO_883 and HipO_976 showed a significantly greater decrease in HipO activity in 81–176 than in M1Cam. Statistics calculated using two-way ANOVA, *** = *p* < 0.001, ** = *p* < 0.01 and * = *p* < 0.05, error bars = SD

Following successful CRISPRi-based repression of *astA*, the gene *hipO* was targeted to further validate the tool. A total of 6 sgRNA target locations from 5’ to 3’ along *hipO* (Fig. 4A) were identified using the sgRNA target generator script and subsequent quality control described previously. The sgRNA target sequences were integrated into CRISPRi plasmids to produce pRC97-pRC102 (Table S2) which were then used to transform M1Cam and 81–176. Following this, transformants were assessed for HipO activity using a hippicurase assay.

The results of the hippicurase assay showed a significant decrease in HipO activity in 81–176 for all sgRNA locations (Fig. 4B). A significant decrease in HipO activity was detected in M1Cam for all sgRNA locations except HipO_883. Furthermore, the level of HipO activity appeared to decrease with sgRNA locations further from the 5’ region, although the pairwise comparisons were not statistically significant. When relative percentage decreases in HipO activity were calculated, this revealed that sgRNAs HipO_676, HipO_883 and HipO_976 showed a greater decrease in HipO activity in 81–176 in comparison to M1Cam (Fig. 4C). This highlighted that there may be inter-strain differences in the efficacy of targeting certain gene locations with CRISPRi-based repression. Overall, these results further validated the functioning of the CRISPRi-based repression tool by demonstrating repression of *hipO* in M1Cam and 81–176.

### Targeting genes within the flagella biosynthesis pathway with the CRISPRi-based repression tool

**Fig. 5:**
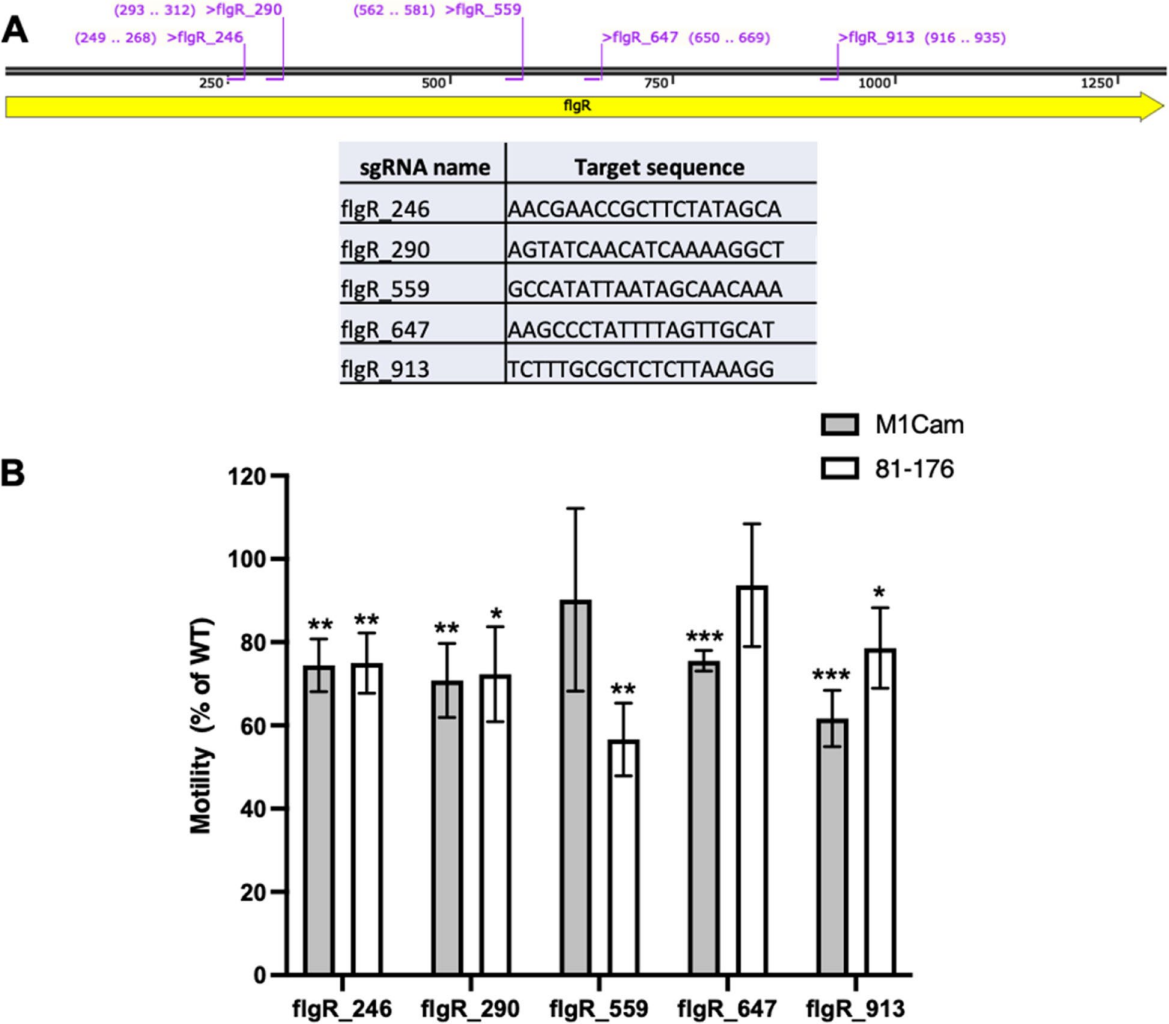
Assessing the impact of CRISPRi-based repression of *flgR* on M1Cam and 81–176 motility. Five sgRNA target locations (A) were identified in *flgR* in M1Cam and 81–176. Motility plating of CRISPRi transformants (B) showed a significant decrease in motility (* = *p* < 0.05, ** = *p* < 0.01 and *** = *p* < 0.001, One-way T-test, N = 3 biological replicates, error bars = SD) for all sgRNA locations except M1Cam flgR_559 and 81–176 flgR_647, which showed no significant difference in motility

**Fig. 6:**
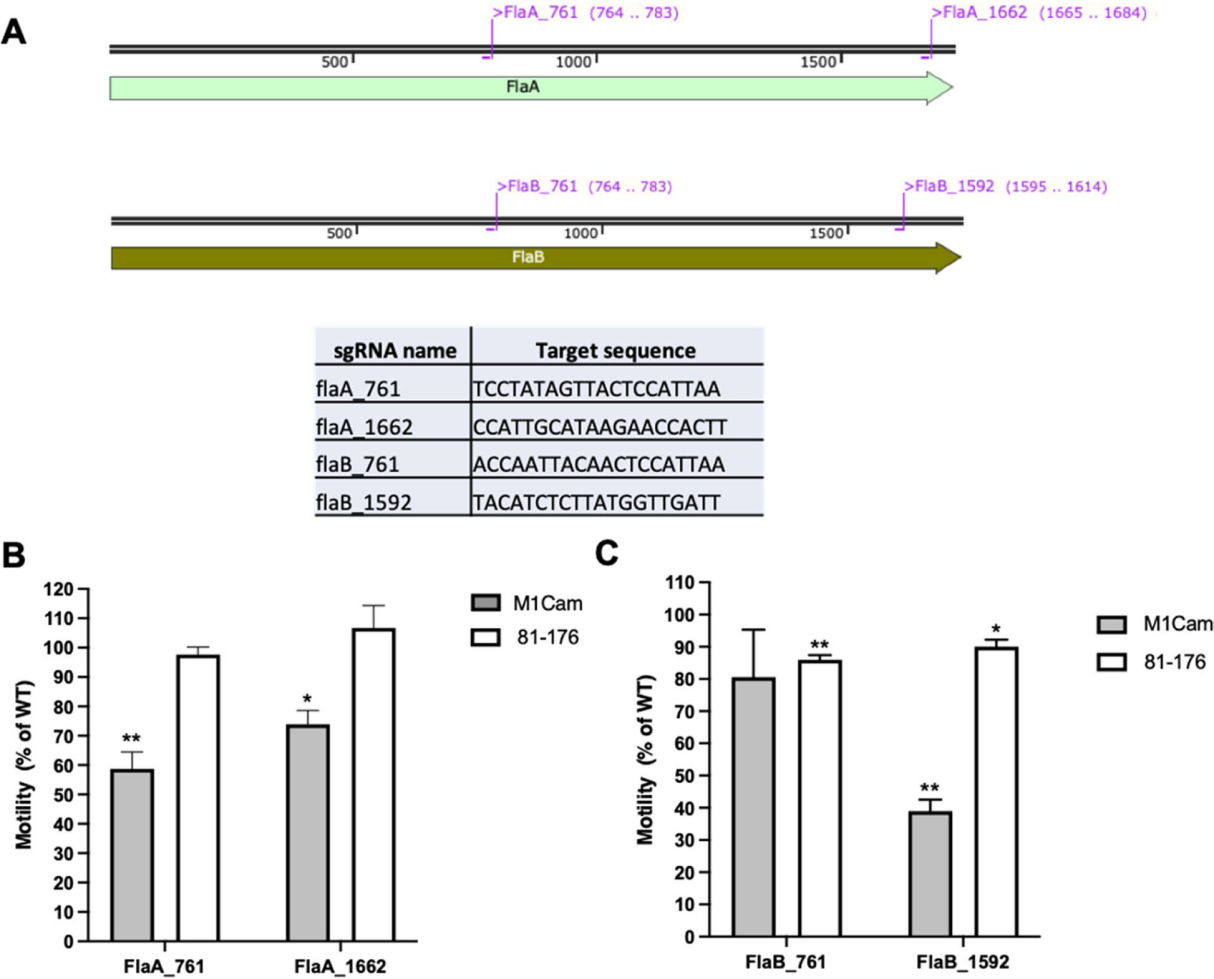
Assessing the impact of CRISPRi-based repression of *flaA* and *flaB* independently in M1Cam and 81–176 motility. Two sgRNA target locations unique to each of *flaA* and *flaB* were identified (A). Motility analysis of CRISPRi transformants targeting *flaA* (B) showed a significant motility reduction in M1Cam flaA_761 and flaA_1662, but no reduction in 81–176 flaA_761 and flaA_1662. Motility plating of CRISPRi transformants targeting *flaB* (C) showed a small but significant reduction in motility in 81–176 flaB_791 and flaB_1592, and a significant decrease in motility in M1Cam flaB_1592 but no significant decrease in M1Cam flaB_1662 (* = *p* < 0.05, ** = *p* < 0.01, One-way T-test, *N* = 3 biological replicates, error bars = SD)

**Fig. 7:**
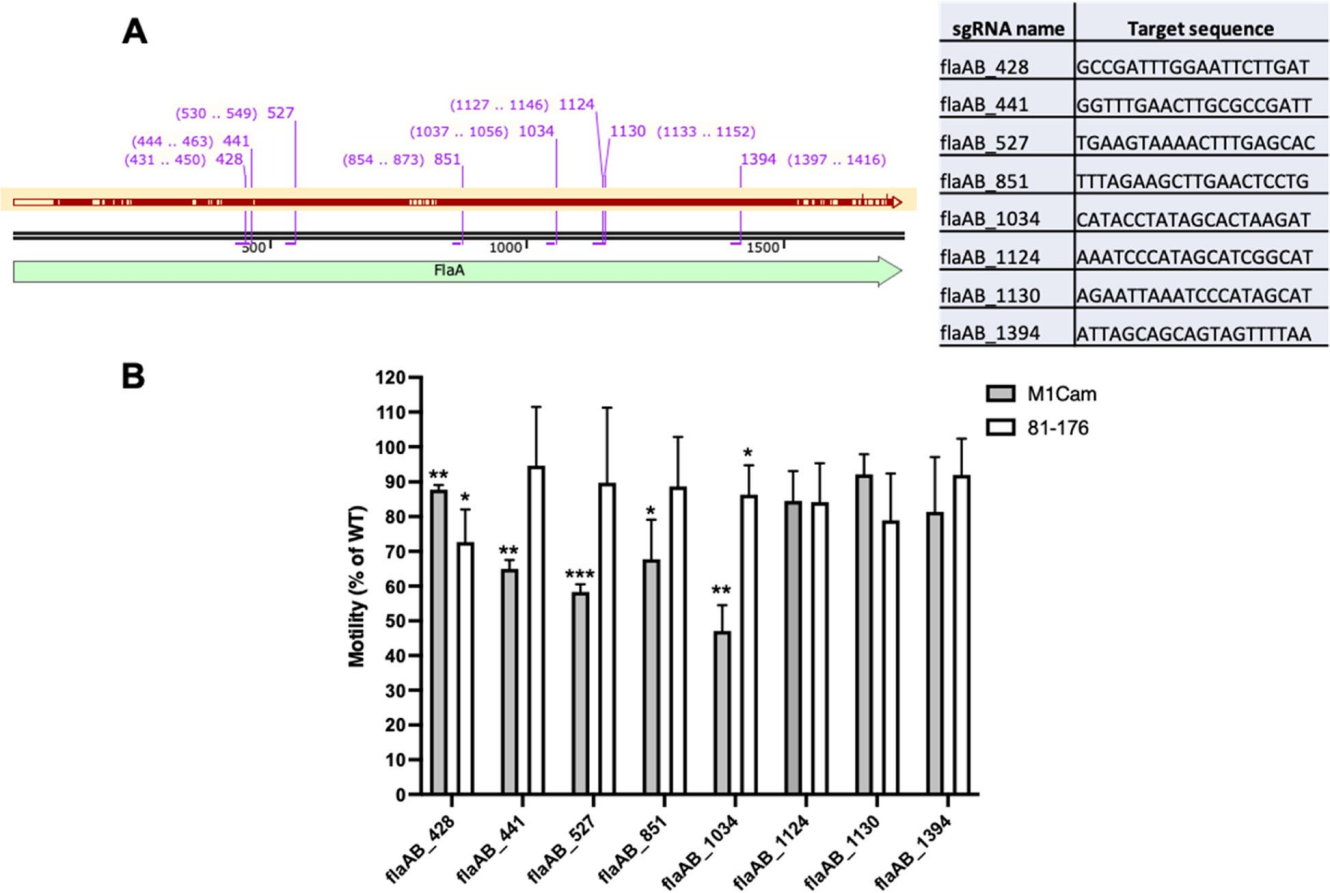
Testing whether *flaA* and *flaB* can be simultaneously targeted by a single sgRNA in M1Cam and 81–176. Eight sgRNA target locations were identified in M1Cam and 81–176 *flaA* and *flaB*, whereby the red arrow represents shared sequence identity between *flaA* and *flaB* (A). Motility plating of CRISPRi transformants targeting *flaA* and *flaB* showed a significant motility decrease in M1Cam flaAB_428, flaAB_441, flaAB_527, flaAB_851 and flaAB_103, and 81–176 flaAB_428 and flaAB_1034 (B) (* = *p* < 0.05, ** = *p* < 0.01 and *** = *p* < 0.001, One-way T-test, *N* = 3 biological replicates, error bars = SD)

The *flgR*, *flaA* and *flaB* gene sequences from M1Cam and 81–176 were screened for sgRNA target sequences using the methods, and selection criteria, described above. This identified 5 sgRNA target sequences in *flgR* (Fig. 5A), 2 in *flaA*, and 2 in *flaB* (Fig. 6A)*.* The low number of sgRNA target sequences found for *flaA* and *flaB* was attributed to the fact that *flaA* and *flaB* share ﻿92.5% nucleotide identity, meaning that shared sgRNA target sequences were discarded to limit off-target effects. This raised the question as to whether sgRNA target locations shared by *flaA* and *flaB* could be used to repress both *flaA* and *flaB* simultaneously. To address this, *flaA* and *flaB* gene sequences were screened for sgRNA target sequences using whole genome sequences omitting *flaB* and *flaA,* respectively. This identified 8 sgRNA target sequences present in both *flaA* and *flaB* in M1Cam and 81–176 (Fig. 7A).

Following this, each of the 17 sgRNA target sequences were used to replace AstA_523 in the CRISPRi plasmid pRC44, to form plasmids pRC56, pRC57, pRC59, pRC61, pRC62, pRC103, pRC104, and pRC106-pRC116 (Table S2). These plasmids were then used to transform WT M1Cam and WT 81–176, resulting in 34 successful transformants. Phenotypic assessment of the motility of each transformant was then carried out using soft-agar motility plating.

In M1Cam and 81–176 CRISPRi transformants targeting *flgR*, there was a significant reduction in motility for all sgRNA target locations (Fig. 5B) except flgR_559 in M1Cam and flgR_647 in 81–176. The pattern of sgRNA target location efficacy differed between strains, with 81–176 flgR_559 displaying the greatest decrease in motility, whilst M1Cam flgR_559 showed no significant reduction in motility. Instead, the sgRNA target location demonstrating the greatest decrease in motility in M1Cam was flgR_913. The sgRNA flgR_647 showed a significant decrease in motility in M1Cam but no significant decrease in 81–176.

Next, M1Cam and 81–176 CRISPRi transformants targeting *flaA* were assessed for motility. Interestingly, no significant decrease in motility was shown for sgRNA target locations flaA_761 and flaA_1662 in 81–176 (Fig. 6B), whilst M1Cam demonstrated a significant decrease in motility for both locations, with flaA_761 showing the greatest reduction (Fig. 6B).

Targeting of *flaB* using sgRNA target locations flaB_761 and flaB_1592 revealed a small but significant reduction in motility in 81–176 (Fig. 6C). In M1Cam, targeting of flaB_761 resulted in no reduction in motility, but targeting of flaB_1592 resulted in a significant decrease in motility (Fig. 6C).

Following these results, M1Cam and 81–176 CRISPRi transformants targeting sgRNA locations present in both *flaA* and *flaB* were assessed for motility (Fig. 7B), to investigate whether both genes could be targeted for repression simultaneously using a single sgRNA. As with the results in targeting *flgR*, *flaA* and *flaB*, sgRNAs targeting *flaA* and *flaB* simultaneously displayed differing efficacies between strains M1Cam and 81–176. M1Cam showed a significant decrease in motility for sgRNA target locations flaAB_428, flaAB_441, flaAB_527, flaAB_851 and flaAB_1034, with the latter exhibiting the greatest reduction in motility (Fig. 7B). Meanwhile, 81–176 demonstrated a significant decrease in motility for sgRNA target locations flaAB_428 and flaAB_1034, with the former showing the largest reduction in motility (Fig. 7B).

Abolition of motility was not achieved in any of the M1Cam and 81–176 CRISPRi transformants targeting *flgR*, *flaA*, *flaB*, or *flaA* and *flaB* simultaneously. Overall, the data showed that the CRISPRi-based repression tool was able to repress the target gene(s) with a resultant decrease in motility, however it was unable to deliver full repression of the target gene(s).

To investigate transcript levels of genes targeted for CRISPRi-based repression quantitative reverse transcription PCR (RT-qPCR) was performed using a selection of CRISPRi transformants that demonstrated the overall greatest decrease in motility in each strain. These included the M1Cam CRISPRi transformants targeting sgRNA sequences flgR_913, flaA_761, flaB_1592 and flaAB_1034, and the 81–176 transformants targeting flgR_559 and flaAB_428.

### RT-qPCR analysis of selected CRISPRi transformants targeting *flgR*, *flaA*, *flaB*, and *flaA/flaB*

RNA was extracted from M1Cam flgR_913, flaA_761, flaB_1592 and flaAB_1034, and 81–176 flgR_559 and flaAB_428. cDNA was generated from the RNA and used for RT-qPCR analysis. For each target gene, primers (Table S3) were designed to amplify two qPCR products, one in a region upstream of the sgRNA target location and one in a region downstream of it. These were notated as the gene name followed by “U” or “D”, whereby “U” was the product upstream the sgRNA target location and “D” was the product downstream of it (*e.g., flgR*_U and *flgR*_D). This was to gain an insight into how the binding of the sgRNA:dCas9 complex affected the transcription upstream and downstream of the target site. For the RT-qPCR analysis of CRISPRi transformants targeting *flgR*, primers were included for *flaA* and *flaB* PCR products to provide an insight of the downstream effects of altered *flgR* expression. The housekeeping gene *gyrA*, was used as a control to normalise experimental cycle thresholds against[8].

**Fig. 8:**
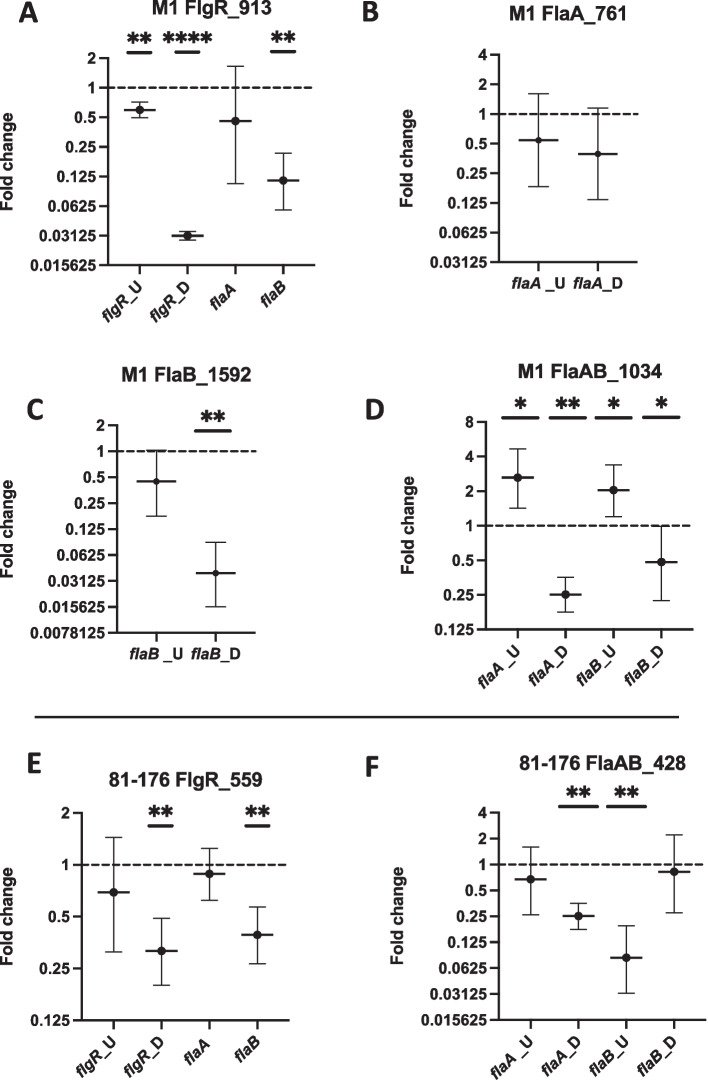
RT-qPCR analysis of selected M1Cam and 81–176 CRISPRi transformants targeting *flgR*, *flaA*, *flaB* and *flaA*/*flaB*. RT-qPCR was performed on M1Cam flgR_913 (A), flaA_761 (B), flaB_1592 (C), flaAB_1034 (D), 81–176 flgR_559 (E) and flaAB_428 (F) to gain an insight into levels of repression elicited in each gene. RT-qPCR products lying upstream (U) and downstream (D) of the sgRNA target locations were designed for each gene, denoted as *gene*_U and *gene*_D respectively. The genes *flaA* and *flaB* were included in RT-qPCR analyses for M1Cam flgR_913 and 81–176 flgR_559 as downstream indicators of the consequences of *flgR* repression. * = *p* < 0.05, ** = *p* < 0.01 and **** = *p* < 0.0001. Statistics calculated using One-way T-tests of ∆∆ct values, *N* = 3 biological repeats, error bars = 95% CI

The RT-qPCR results from M1Cam flgR_913 showed a significant decrease in *flgR*_U, *flgR*_D and *flaB* products in comparison to M1Cam (Fig. 8A). *flgR*_D demonstrated the greatest fold change of 0.03, equating to ~ 33.3-fold decrease, in comparison to *flgR*_U which displayed a fold change of 0.59, equating to ~ 1.6-fold decrease. This indicated greater repression of *flgR* downstream of the sgRNA:dCas9 binding site at flgR_913 compared to the region upstream of the site. No significant reduction in *flaA* was detected compared to M1Cam, suggesting *flaA* transcription was not significantly reduced following the detected repression of *flgR*. However, *flaB* showed a significant fold change of 0.11, equating to ~ 8.8-fold decrease.

RT-qPCR analysis of M1Cam flaA_761 showed no significant fold change in *flaA*_U or in *flaA*_D compared to M1Cam (Fig. 8B). Meanwhile, M1Cam flaB_1592 demonstrated a significant negative fold change for *flaB*_D of 0.04, equating to ~ 25.6-fold decrease (Fig. 8C). However, there was no significant fold change for *flaB*_U. As the sgRNA:dCas9 binding site at flaB_1592 lies 139 bp from the 3’ end of the gene (*flaB* is 1731 bp long), these results suggest that the beginning of the *flaB* transcript was transcribed but potentially stopped short at flaB_1592.

The results for M1Cam flaAB_1034 showed significant fold changes for *flaA*_U, *flaA*_D, *flaB*_U and *flaB*_D (Fig. 8D). Interestingly, both *flaA*_U and *flaB*_U displayed ~ 2.6 and ~ 2.0-fold increase respectively. Meanwhile *flaA*_U and *flaB*_U demonstrated 0.25 and 0.48-fold changes, equating to ~ 4.0 and ~ 2.1-fold decreases respectively. As expected, this indicated that the region upstream of the sgRNA:dCas9 binding site at flaAB_1034 in *flaA* and *flaB* was transcribed to a greater level in comparison to M1Cam, whilst the region downstream was transcribed to a lower level.

In 81–176 flgR_559, the RT-qPCR analysis showed no significant fold change in *flgR*_U, but demonstrated a significant negative fold change of 0.32 for *flgR*_D, equating to ~ 3.1-fold decrease (Fig. 8E). Like the results for M1Cam flgR_913, no significant fold change was exhibited in *flaA*, but a significant negative fold change of 0.39 was displayed in *flaB*, equating to ~ 2.6-fold decrease.

Finally, RT-qPCR analysis of 81–176 flaAB_428 showed significant negative fold changes of 0.25 and 0.08 in *flaA*_D and *flaB*_U, equating to ~ 4.0 and ~ 12-fold decreases respectively (Fig. 8F). No significant fold changes were demonstrated in *flaA*_U and *flaB*_D. This indicated that for *flaA*, targeting of flaAB_428 resulted in significant repression downstream of the sgRNA target location, but not upstream of it. Conversely, for *flaB,* targeting of flaAB_428 resulted in significant repression upstream of the sgRNA target location, but not downstream of it.

Overall, the RT-qPCR analysis showed that the CRISPRi-based repression tool elicited varying levels of repression of target genes in CRISPRi strains M1Cam flgR_913, flaB_1592, flaAB_1034, 81–176 flgR_559 and flaAB_428. No repression of *flaA* was detected in M1Cam flaA_761.

It is worth noting that the motility assays and RT-qPCR reported a mean value which was representative of the whole population of bacteria used. Therefore, this did not account for the possibility of population heterogeneity which has been reported in *C. jejuni*[47],whereby complete repression may have occurred in some bacteria but not others. To investigate the phenotype of each population and assess the possibility of sub-populations, TEM was used to visualise populations of bacteria from each CRISPRi transformant population, to identify whether any population-level heterogeneity was observed, for example bacteria with flagella and bacteria without flagella.

### TEM analysis of selected CRISPRi transformants targeting *flgR*, *flaA*, *flaB*, and *flaA/flaB*

TEM analysis of M1Cam flgR_913, flaA_761, flaB_1592 and flaAB_1034, and 81–176 flgR_559 and flaAB_428 was performed to provide a gross visual overview of phenotypic presentation of flagella within each population. This was to ascertain whether there was a consistent phenotype within each population, or whether there were patterns of phenotypic heterogeneity – for example, aflagellate bacteria and fully flagellated bacteria. This was partly motivated by previous studies that have identified that WT *C. jejuni* can display within-population phenotypic heterogeneity[47, 49, 50].

**Fig. 9:**
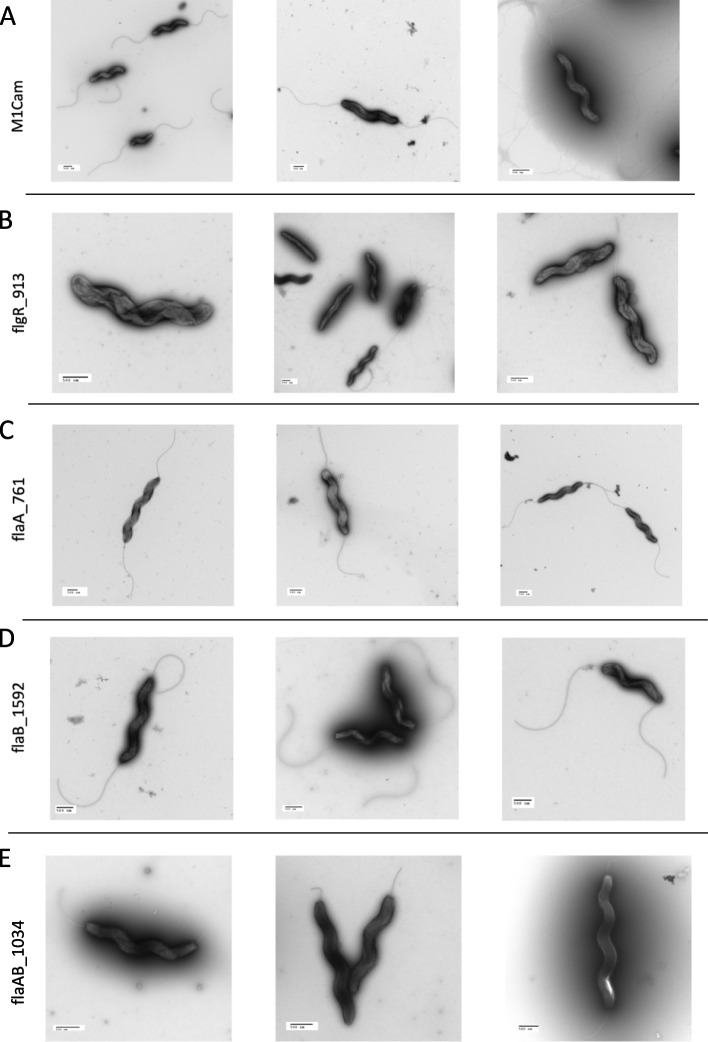
TEM visualisation of M1Cam CRISPRi transformants targeting *flgR*, *flaA*, *flaB* and *flaA*/*flaB*. TEM analysis showed that CRISPRi transformants M1Cam flgR_913 (B), and flaAB_1034 (E) showed different phenotypes in comparison to M1Cam (A). M1Cam flgR_913 showed an aflagellate phenotype with some individuals exhibiting bipolar flagella (B). M1Cam flaA_761 (C) an M1Cam flaB_1592 (D) showed no difference in phenotype compared to M1Cam, and M1Cam flaAB_1034 showed short bipolar or single flagella (E). TEM images displayed were selected as representative examples of images taken from 3 biological replicates. Scale bar = 500 nm

TEM of in vitro grown WT M1Cam bacteria demonstrated a phenotype with bipolar flagella, which served as a baseline for comparison of M1Cam CRISPRi transformants (Fig. 9A). In contrast, the M1Cam flgR_913 populations showed a variety of phenotypes (Fig. 9B). Predominantly, an aflagellate phenotype was observed, with occasional individual bacteria showing short to mid-length single or bipolar flagella, and some with full-length bipolar flagella. The M1Cam flaA_761 populations contained bacteria with full-length flagella (Fig. 9C). Similarly, M1Cam flaB_1592 bacteria appeared extremely similar to M1Cam bacteria, with full-length bipolar flagella (Fig. 9D). Finally, the M1Cam flaAB_1034 bacteria grossly exhibited short bipolar flagella, often with one slightly longer flagellum (Fig. 9E).

**Fig. 10:**
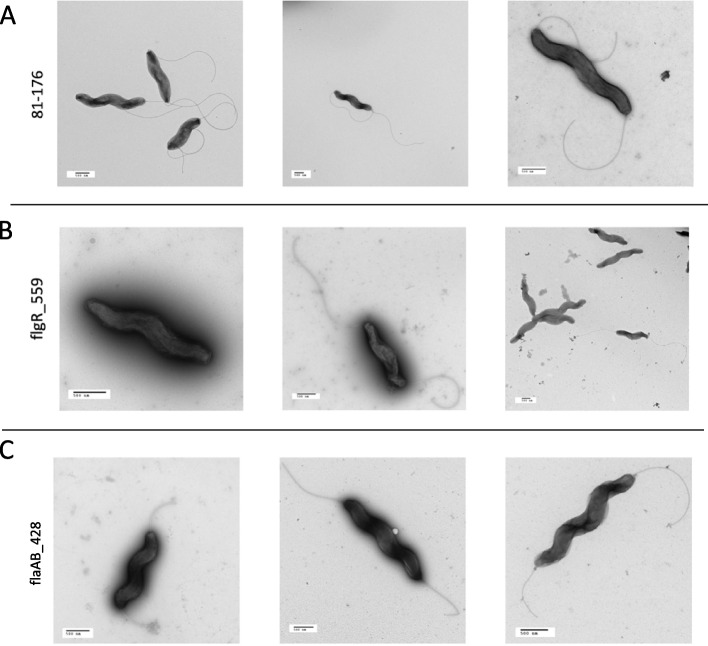
TEM visualisation of 81–176 CRISPRi transformants targeting flgR and flaA/flab. TEM analysis showed that CRISPRi transformants 81–176 flgR_559 (B) and 81–176 flaAB_428 (C) displayed different phenotypes in comparison to 81–176 (A). 81–176 flgR_559 grossly demonstrated an aflagellate phenotype with some bacteria exhibiting bipolar flagella (B). 81-176_flaAB428 demonstrated short to mid-length flagella (C). TEM images displayed were selected as representative examples of images taken from 3 biological replicates. Scale bar = 500 nm

TEM of in vitro grown WT 81–176 bacteria showed bipolar flagella against which 81–176 CRISPRi transformants were compared (Fig. 10A). The 81–176 flgR_559 bacteria showed a similar distribution of phenotypes to M1Cam flgR_913, with most of the bacteria visualised displaying an aflagellate phenotype, with infrequent observation of a few bacteria with short to mid-length single or bipolar flagella, and some bacteria with full-length bipolar flagella (Fig. 10B). The 81–176 flaAB_428 bacteria showed short to mid-length bipolar flagella (Fig. 10C).

## Discussion

Taken together, the results demonstrate that the *S. pyogenes*-derived CRISPRi repression tool developed here with selected sgRNA target sequences could elicit effective repression of the biosynthetic genes *astA* and *hipO* in *C. jejuni* strains M1Cam and 81–176. The results also showed that the CRISPRi-based repression tool could be applied to genes within the complex flagella biosynthetic pathway, resulting in varying levels of motility reduction and flagella phenotypes.

When developing the CRISPRi-based repression tool, it was necessary to carefully consider the levels at which dCas9 and the sgRNAs were expressed. Previously it has been shown that overexpression of *dcas9* in combination with sgRNAs containing one of a series of specific sequences in the first 5 nucleotides proximal to the PAM (known as a “bad seed” sequence) can be toxic in bacterial cells[30]. Conversely, insufficient levels of dCas9 and sgRNAs result in low repression efficacy[51]. Balancing these requirements is essential to form a functioning CRISPRi tool. Promoter screening using a LacZ reporter assay provided a valuable insight into promoter activity which informed the decision to use pCat and pPorA for dCas9 and sgRNA expression respectively. Whilst the combination of pCat and pPorA in expressing *dcas9* and sgRNA respectively resulted in successful *astA* and *hipO* repression with selected sgRNA target sequences, the trial of other promoters in the CRISPRi-based repression tool could allow a range of repression levels. For instance, pCat could be swapped with a less active promoter such as pMetK in driving dCas9 and therefore elicit a lower level of repression. Indeed, promoters outside of the four used in this study could also be assessed for their activity and be used to drive *dcas9* expression to achieve a range of repression levels. Finally, the addition of an inducible promoter driving *dcas9* expression would allow tuning of repression through alteration of cellular dCas9 levels, which has been used previously in pooled CRISPRi screens[10, 52], and in functional analysis of genes of interest[53].

The location of sgRNA targets across the length of the *astA* or *hipO* genes from 5’ to 3’ did not appear to have a significant impact on the efficacy of the system. Aside from *dcas9* and sgRNA expression levels, the location of regions targeted by sgRNAs has been shown to be highly important in CRISPRi design[9, 54]. Concentration of targets at the 5’ end of the gene (within the first 5% of the open reading frame) have typically been favoured due to the formation of a transcriptional road block by the CRISPRi complex before most of the gene has been transcribed[9, 45]. To determine if this held true in *C. jejuni*, a range of sgRNAs targeting from 5’ to 3’ in *astA* and *hipO* were evaluated. The results of the CRISPRi repression of *astA* showed that there was no significant difference between each of the sgRNAs used along the length of the gene in both M1Cam and 81–176. When targeting *hipO* there was also no significant difference between sgRNA location in M1Cam and 81–176.

Although sgRNA target location did not have a significant effect on the repression of *astA* and *hipO*, some sgRNA target locations demonstrated significantly different repression efficacies between strains. For example, comparison of relative decreases in AstA activity between M1Cam and 81–176 showed that sgRNAs astA_85 and astA_1091 demonstrated a significantly greater efficacy in 81–176 than M1Cam. An equivalent comparison between HipO activity decreases showed that sgRNAs HipO_676, HipO_883 and HipO_976 also showed a significantly greater efficacy in 81–176 than M1Cam. This highlights the importance of validating results using multiple strains, if practical to do so.

Another key element in this validation work was the comparison of the application of CRISPRi-based repression in M1Cam and the CRISPR-Cas9 region deletion mutant M1Cam∆CRISPR. Generation of the mutant enabled comparison of CRISPRi function in both strains, in order to detect any competition between the endogenous CRISPR-Cas9 system in M1Cam and the *S. pyogenes*-derived CRISPRi components. Competition between these elements could potentially affect CRISPRi repression efficacy, and therefore had to be considered. For instance, one theoretical example of competition could be the interaction of *S. pyogenes* sgRNA with the endogenous *C. jejuni* Cas9 protein. This could potentially deplete the amount of *S. pyogenes* sgRNA available for complexing with *S. pyogenes* dCas9 and thereby affect repression efficacy. Moreover, should interaction between *S. pyogenes* sgRNA and *C. jejuni* Cas9 occur, this could result in genomic DNA cleavage at sgRNA target sites, although this would require the presence of appropriate *C. jejuni* PAM recognition sites. Another theoretical example of competition could be between the *S. pyogenes* sgRNA and endogenous *C. jejuni* tracrRNA:crRNA complex. If the *C. jejuni* tracrRNA:crRNA complex was able to interact with the *S. pyogenes* dCas9 protein, this would lead to reduced dCas9 interaction with the *S. pyogenes* sgRNA and potentially lower CRISPRi repression efficacy.

Despite these potential sources of competition, the nitrophenol assay results showed no detectable difference in the CRISPRi repression efficacy between M1Cam and M1Cam∆CRISPR. No significant differences between relative percentage decrease in AstA activity were detected between M1Cam and M1∆CRISPR for each sgRNA location. This suggested that any potential competition between the systems did not detectably interfere with the repression capability of the CRISPRi tool.

The reason why no difference was found could be because *S. pyogenes* and *C. jejuni* CRISPR-Cas9, although both being Type-II CRISPR systems, have some fundamental differences. For example, X-ray crystallography analysis has shown that *C. jejuni* sgRNA forms a unique triple helix structure when in complex with *C. jejuni* Cas9, which has not been observed in *S. pyogenes* sgRNA:Cas9 interactions[55]. This means that the *C. jejuni* tracrRNA:crRNA complex may not be able to successfully interact with *S. pyogenes* Cas9. Secondly, the *C. jejuni* and *S. pyogenes* Cas9 proteins each have a different PAM recognition motif (5’-NGG-3’ and 5’-NNNVRYM-3’ respectively)[55], indicating that if either the *S. pyogenes* dCas9 or the *C. jejuni* Cas9 could harness tracrRNA:crRNA or sgRNAs respectively, it is unlikely that the complexes would be able to bind to target DNA effectively.

Following validation of the CRISPRi-based repression tool against *astA* and *hipO*, the tool was used to target the genes *flgR*, *flaA*, *flaB*, and both *flaA* and *flaB* (simultaneously) within the highly complex flagella biosynthesis pathway. Soft-agar motility plating provided a convenient means to assess the phenotypic impact of targeting each gene(s). Following this, a selection of CRISPRi transformants were examined in-depth using RT-qPCR to gain an insight into the expression of genes targeted for repression. Furthermore, TEM analysis was used to provide an overview of the gross phenotypic presentation of each selected CRISPRi transformant.

Taken together, these results provided the opportunity to observe broad patterns in the application of CRISPRi-based repression. Firstly, at the population level, no complete abolition of motility was observed during targeting of each gene, although significant reductions in motility were achieved during CRISPRi-based repression of all genes except for *flaA* in 81–176. Secondly, the results reinforced the observations made during CRISPRi-based repression of *hipO* that there are between-strain differences in sgRNA target location efficacy. This pattern was particularly profound in this set of experiments, whereby the most effective sgRNA target location differed between M1Cam and 81–176 for all genes that showed a reduction in motility (*flgR*, *flaA*, *flaB*). Finally, TEM analysis of selected CRISPRi transformant populations indicated the possibility of within-population variance in the efficacy of CRISPRi-based repression, whereby phenotypes appeared to vary between bacteria in the population.

In addition to broad observations, insights into the outcomes of targeting each gene using CRISPRi-based repression were made. Firstly, targeting of *flgR* demonstrated that the greatest reduction in motility was achieved using sgRNA locations flgR_913 and flgR_559 in M1Cam and 81–176 respectively. Previous studies have indicated that deletion of *flgR* results in an aflagellate, non-motile phenotype[28, 33, 40], therefore the reduction rather than complete abolition of motility in CRISPRi targeting of *flgR* in M1Cam and 81–176 indicated that full repression, within the total population of bacteria, was not being achieved. To interrogate this further, RT-qPCR was performed using RNA extracted from M1Cam flgR_913 and 81–176 flgR_559, and TEM analysis was carried out on populations of each.

The RT-qPCR results for M1Cam flgR_913 showed that *flgR* expression was significantly decreased, with a significant reduction in qPCR products upstream and downstream of the sgRNA:dCas9 binding site at flgR_913. The results also showed a significant decrease in *flaB* expression. As a flagella Class II gene, *flaB* expression is driven by σ^54^, whose transcriptional activity is regulated by the FlgSR system and FlhF[34, 39]. This indicates that the decrease in *flgR* expression could have resulted in a decrease in flagella Class II gene expression (including *flaB*) through this pathway. Interestingly, *flaA* expression was not significantly decreased. As a flagella Class III gene, *flaA* expression is driven by σ^28^, which is itself a flagella Class II gene[34]. If flagella Class II genes had decreased expression, then a decrease in *flaA* expression may have been expected as a result. Phenotypic analysis of M1Cam flgR_913 using TEM provided further context for the motility and RT-qPCR results. TEM imaging broadly showed that the M1Cam flgR_913 population had an aflagellate phenotype, with occasional individual bacteria displaying mid- to full-length single or bipolar flagella. During soft-agar motility plating, these sub-populations may have grown and contributed to the motility measured on the plates. Furthermore, as the RT-qPCR results were an overall (*i.e*., averaged) representation of expression of *flgR*, *flaA,* and *flaB* in the entire population, it is possible that these sub-populations may have had little to no reduction in expression of these genes, which could have contributed to the expression measured.

The RT-qPCR results for 81–176 flgR_559 showed a similar pattern to M1Cam flgR_913. However, there was no significant decrease in expression upstream of the sgRNA:dCas9 binding site at flgR_559, only downstream. This indicated that *flgR* expression was significantly decreased following the sgRNA target sequence location. Expression of *flaB* was significantly decreased, whilst *flaA* expression was not. TEM analysis of 81–176 flgR_559 showed that like M1Cam flgR_913, the predominant phenotype was aflagellate, with fewer mid- to full-length single or bipolar flagella. The presence of these sub-populations may have contributed to the motility and RT-qPCR results recorded for 81–176 flgR_559 as described above for M1Cam. This indicated that the CRISPRi-based repression of *flgR* in both strains resulted in a similar outcome – formation of aflagellate and partial to fully flagellated sub-populations. This population heterogeneity was an important observation, as it indicated that CRISPRi transformant populations should not be assumed to always exhibit fully uniform phenotypes. This phenomenon has also been described in CRISPRi studies in *E. coli* [56].

Next, targeting of *flaA* for CRISPRi-based repression showed that only M1Cam CRISPRi transformants demonstrated a significant reduction in motility. M1Cam flaA_761 demonstrated the greatest decrease in motility, but no abolition of motility (as assessed using motility agar). Typically, deletion of *flaA* results in a non-motile phenotype with a short flagella comprising FlaB[57]. This indicated that full repression of *flaA* expression was not occurring. RT-qPCR using RNA extracted from M1Cam flaA_761 showed no significant decrease in *flaA* expression, and TEM analysis of M1Cam flaA_761 populations showed full length bipolar flagella. Overall, this indicated that despite the observed decrease in motility, significant repression of *flaA* was not detected by RT-qPCR and the TEM analysis showed no reduction in flagella length. Whilst the flagella seemed to be produced, it is possible that the FlaA proteins were truncated, which in turn could have impacted flagellum structure and movement. Neither 81–176 flaA_761 nor flaA_1662 showed a motility decrease in comparison to WT 81–176. This highlighted that on top of inter-strain differences in optimal sgRNA target sequence locations, there are inter-strain differences as to whether a gene can be repressed at all.

Interestingly, 81–176 and M1Cam CRISPRi transformants targeting *flaB* displayed differing motility results. 81–176 flaB_761 and 81–176 flaB_1592 demonstrated a small reduction in motility, whilst M1Cam flaB_761 showed no reduction and M1Cam flaB_1592 exhibited a large decrease in motility. Reduction but no abolition of motility has been reported following deletion of *flaB*, with full length flagella comprising FlaA[57]. RT-qPCR of M1Cam flaB_1592 showed a significant decrease in *flaB* expression downstream of the sgRNA target location site, but no decrease upstream of the site. The location at flaB_1592 was fairly close to the 3’ end of the gene, as *flaB* is 1731 bp, indicating that the RT-qPCR could suggest that the full transcript may not have been produced. The TEM analysis showed that the M1Cam flaB_1592 population had full-length bipolar flagella. This indicated that motility can still be impacted despite the presence of full-length bipolar flagella. Like M1Cam flaA_761, despite the presence of full flagella, it is possible that the FlaB protein could be truncated, thereby impacting the flagellum structure and movement.

The shared sequence identity of *flaA* and *flaB* allowed the opportunity to trial sgRNA to target sequences present in both genes, to determine if a single sgRNA could be used to target two genes for repression at the same time. Targeting of both *flaA* and *flaB* in M1Cam revealed that sgRNA target location flaAB_1034 showed the greatest decrease in motility, however no abolition of motility was achieved. RT-qPCR showed that *flaA* and *flaB* expression was significantly decreased downstream of the sgRNA:dCas9 binding site. This implied that both *flaA* and *flaB* were targeted at population level, although heterogeneity in *flaA* and *flaB* targeting at a single-cell level, cannot be ruled out. Interestingly, *flaA* and *flaB* expression was significantly increased upstream of the sgRNA:dCas9 binding site. TEM analysis of M1Cam flaAB_1034 populations showed that the prevailing phenotype was the presence of short bipolar flagella. This indicated incomplete formation of the flagella, which may have been due to reduced levels of FlaA and FlaB. Alternatively, incomplete flagella may have been due to truncation of FlaA and FlaB, resulting in incorrect filament structure. The shorter flagella were likely a factor in the observed decrease in motility.

Simultaneous targeting of *flaA* and *flaB* in 81–176 revealed that sgRNA target sequence flaAB_428 showed the greatest decrease in motility. RT-qPCR of 81–176 flaAB_428 showed a significant decrease in *flaA* expression downstream of the sgRNA:dCas9 binding site, but not upstream, whilst *flaB* expression was decreased upstream of the binding site but not downstream. This indicated that *flaB* may have been incompletely transcribed – for instance, transcription could have been resumed following the sgRNA:dCas9 binding site, although a within-gene promoter was not identified in the *flaB* sequence[58]. Nevertheless, these findings highlighted that despite targeting identical sgRNA target sequences within *flaA* and *flaB*, the outcome resulted in different patterns of repression. It is also worth considering that although two genes were targeted, the sgRNA expression level was not increased, meaning that the same amount of sgRNAs used previously in these experiments to target one gene were shared across two genes. This in turn may have resulted in incomplete, or lower, repression for each gene.

TEM analysis of 81–176 flaAB_428 bacteria demonstrated a gross phenotype of short- to mid-length bipolar flagella, which may have been a result of reduced FlaA and FlaB levels, or truncated FlaA and FlaB proteins, leading to incomplete flagella formation. This in turn likely contributed to the decrease in motility observed. Taken together, the targeting of *flaA* and *flaB* in M1Cam and 81–176 using a single sgRNA target sequence showed that it is possible to target two genes with shared sequence identity for repression simultaneously.

Overall, we have demonstrated that an *S. pyogenes*-derived CRISPRi-based repression tool can be integrated onto the chromosome of two *C. jejuni* strains and be used to repress the expression of the target genes *astA* and *hipO*. Whilst the presence of a CRISPR-Cas9 system in M1Cam could have been a factor in CRISPRi repression efficacy, deletion of it did not elicit any significant detectable difference in CRISPRi repression. We have also demonstrated that genes within the flagella biosynthetic pathway (*flgR*, *flaA*, *flaB* and *flaA/flaB*) can be targeted using the CRISPRi-based repression tool. This is the first report of a CRISPRi-based repression tool in *C. jejuni* and provides a basis for future application and development as a highly versatile molecular tool for the regulation of gene expression. We believe that it will be a valuable addition to the *Campylobacter* molecular-toolbox to better understand this important pathogen, with the aim of identifying means to reduce colonisation of food-producing animals and to treat or prevent infection in humans and other animals.

## Materials and methods

### Bacterial strains and growth conditions

All *C. jejuni* strains were cultured on Mueller–Hinton (MH) agar (Oxoid) supplemented with appropriate antibiotics and substrates. All *C. jejuni* strains were grown at 42 °C under microaerophilic conditions (5% O_2_, 5% CO_2_, 5% H_2_ and 85% N_2_) in a M95 Variable Atmosphere Workstation (Don Whitley, Shipley, United Kingdom). For long term storage, bacterial strains were scraped into Microbank (ProLab Diagnostics) cryovials using 1 μl inoculation loops and stored at -80 °C. *C. jejuni* strains from freezer stocks were resuscitated by streaking onto MH agar using 1 μl inoculation loops, followed by growth for 48 h. Bacterial growth was then re-streaked onto MH agar plates and harvested for use after 16 h, by scraping into MH broth using an L-shaped spreader. Bacterial strains used in this study are detailed in Table S1.

### Plasmids and primers

Plasmids generated in this study are detailed in Table S2. Primers used in this study are detailed in Table S3.

### Molecular biology

All PCRs to generate *lacZ* reporter constructs used Q5 High Fidelity Polymerase (New England Biolabs). A plasmid (pSV009) that we have previously reported for homologous recombination[25] (Table S2) was used as a template vector in the design of all *lacZ* reporter constructs. pSV009 features two 400 bp homologous flanking arms to the M1Cam pseudogenic region CJM1_0055– 0057 which has > 98% sequence identity in 81–176. pSV009 was modified to remove a pCat promoter and add a MluI site, yielding pRC1 (Table S2).

Primers ES02, RC20, RC118 and RC129 were used with primer RC18 (Table S3) to amplify and fuse the promoters pPorA, pCat, and pMetK to the *lacZ* gene amplified from *E. coli* K12 genomic DNA. Primers RC21 and RC18 (Table S3) were used to amplify the *lacZ* gene without a promoter. PCR products were digested with XhoI and BamHI (New England Biolabs) and ligated with pRC1 to produce pRC2, pRC3, pRC27 and pRC28 (Table S2).

### Selection of target sequences and sgRNA design

The DNA sequences for each target gene in M1Cam and 81–176 (Genbank accession numbers: CP012149.1 and CP022551.1, respectively) were downloaded from the NCBI database (https://www.ncbi.nlm.nih.gov/nucleotide/). The Python script sgRNA_design_main.py[45] was used to generate a list of sgRNA target sequences for each gene. The lists were then cross-referenced, and only sgRNA target sequences that appeared in both M1Cam and 81–176 lists were taken forward. These sequences were then entered into the Python script gRNAInteractionScreen_ThymeFeb2016 2.py to omit sequences containing hairpin formation with the sgRNA backbone[46]. Primers were designed for integration of sgRNA target sequences into sgRNA plasmids (Table S2). DNA construct and sgRNA target illustrations were prepared using SnapGene Viewer (GSL Biotech; available at snapgene.com).

### Construction of CRISPRi plasmids

An sgRNA expression construct was designed in silico, to include a pPorA sequence, sgRNA target sequence astA_47, a sgRNA scaffold from *S. pyogenes* and a terminator sequence and ordered as a GeneArt Strings DNA Fragment (Thermofisher), which was digested with BamHI and PstI and ligated with pRC1 to produce pRC35. This plasmid was used as a template for inverse PCR to create all other sgRNA plasmids (Table S2). The *dcas9* sequence was amplified from the plasmid pdCas9 (Addgene plasmid # 46,569) using primers RC133 and RC135 (Table S3) to fuse the pCat sequence to *dcas9*. The product was digested with XhoI and AgeI and ligated into each respective sgRNA plasmid to produce all CRISPRi plasmids (Table S3).

### Transformation of *C. jejuni* strains

Natural transformation of *C. jejuni* was performed using an adapted biphasic method[59, 60] with EcoRI-methylated DNA[61]). Resuscitated bacteria were scraped into 1 ml MH broth using an L-shaped spreader, diluted to an OD_600nm_ of 0.5 and 500 μl was added on top of 2 ml of MH agar with 5% (v/v) defibrinated horse blood (Oxoid) ​​in a 30 ml universal tube (Greiner Bio-One). Cells were then incubated at 42 °C under microaerophilic conditions for 3 h. 300 ng methylated DNA was added to the bacteria in 20 μl dH2O, which were then incubated at 42 °C under microaerophilic conditions for 3-5 h, followed by selection plating on MH-Blood plates supplemented with appropriate antibiotics, and growth in microaerophilic conditions for 3–5 days.

### Construction of M1Cam CRISPR array mutant M1Cam∆CRISPR

The CRISPR locus targeted for deletion spanned a 4965 bp region containing the CRISPR array, *cas2*, *cas1,* and *cas9* (M1Cam genome location 1,431,507–1,436,472 GenBank: CP012149.1). The CRISPR locus deletion construct was designed in silico by adding ~ 500 bp sequence flanking either side of the CRISPR locus to a chloramphenicol acetyltransferase *cat* resistance cassette[25] to yield a homologous recombination construct. NdeI and MfeI restriction sites were added at the 5’ and 3’ end and the construct ordered as a GeneArt (Thermofisher) plasmid. The plasmid was digested with NdeI and MfeI and the construct was gel-extracted before ligation into gel-extracted pRC1 digested with NdeI and MfeI. Ligations were transformed into chemically competent *E. coli* DH5α cells (New England Biolabs) followed by selection-plating on LB-Cm-Amp agar plates. This yielded pRC_CRISPR_Del (Table S2). M1Cam CRISPR locus deletion strains were PCR verified using primers RC182 and RC183 (Table S3). Sanger sequencing of homologous recombination sites using primers RC182 and RC183 was performed to check for scar-less recombination (Table S3).

### RNA extraction and RT-qPCR

*C. jejuni* strains were grown in liquid culture for 16 h or until the OD_600nm_ reached 0.4. 2 ml RNAprotect Bacteria Reagent (Qiagen) was added to 1 ml of each culture in a 15 ml Falcon tube (Greiner), vortexed and incubated for 5 min at room temperature. Tubes were centrifuged for 10 min at 5000 × *g*, and the supernatant decanted. RNA extraction was performed on pellets using a Qiagen RNeasy mini kit according to the manufacturer’s protocol.

Reverse transcription was carried out using LunaScript RT SuperMix Kit (New England Biolabs). 1 μg of RNA was added to a 20 μl reaction containing a final concentration of 1 × LunaScript RT SuperMix. In parallel, 1 μg of RNA was added to a 20 μl reaction containing a final concentration of 1 × No RT control mix. Reactions were incubated in a thermocycler at 25 °C for 2 min, 55 °C for 10 min and 95 °C for 1 min.

qPCR was performed using 3 biological replicates in triplicate using Luna Universal qPCR Master Mix (New England Biolabs). The gene *gyrA* was used as a housekeeping gene[8]. 1 μl cDNA was added to a 20 μl reaction containing a final concentration of 1 × Luna Universal qPCR Master Mix, and 0.25 μM of each primer. No-RT control samples were included to check for gDNA contamination, along with negative controls using water. The qPCR was performed using a RotorGene Q (Qiagen) using the SYBR setting. Thermocycling conditions included an initial denaturation at 95 °C for 1 min, denaturation at 95 °C for 15 s, extension at 60 °C (45 cycles), and melt curve from 60–95 °C. Following every qPCR run, melt curves were analysed to verify the presence of a single curve for each reaction. Cycle threshold (CT) values were recorded and ∆∆CT values calculated using the following equations:

WT ∆CT = WT Ct (gene of interest) – WT Ct (housekeeping gene).

CRISPRi ∆CT = CRISPRi Ct (gene of interest) – CRISPRi Ct (housekeeping gene).

∆∆CT = (WT ∆CT) – (CRISPRi ∆CT).

Analysis of ∆∆CT values was carried out using previously described methods[62]. ∆∆CT values from each biological replicate were used to calculate a mean ∆∆CT and 95% confidence interval (CI) for each gene. A One-way T-test was performed to determine significance of each ∆∆CT value. Fold changes were then calculated for graphical representation, whereby fold change = 2^−∆∆CT^.

### β-galactosidase assays

The β-galactosidase assay protocol was adapted from previously described methods[44, 63]. Resuscitated *C. jejuni* strains were harvested into 1 ml MH broth using an L-shaped spreader and diluted to an OD_600nm_ of 0.2, which was used to prepare a starter culture of OD_600nm_ of 0.005 in MH broth. Cultures were grown in microaerophilic conditions at 200 RPM at 42 °C for 16 h or until the OD_600nm_ reached 0.4. 40 μl culture was removed and added to 60 μl permeablisation solution (100 mM Na_2_HPO_4_, 20 mM KCl, 2 mM MgSO_4_, 0.8 mg/ml hexadecyltrimethylammonium bromide, 0.4 mg/ml sodium deoxycholate, 5.4 μl/ml beta-mercaptoethanol) and incubated at 30 °C for 30 min. 600 μl substrate solution (60 mM Na_2_HPO_4_, 40 mM NaH_2_PO4, 1 mg/ml o-nitrophenyl-β-D-Galactoside (ONPG), 2.7 μl/ml β-mercaptoethanol) was added and the time taken for the mixture to develop a pale-yellow colour was recorded. 700 μl stop solution (1 M Na_2_CO_3_) was added and the mixture centrifuged at 20,000 × g for 1 min. 1 ml reaction mixture was carefully removed and OD_420nm_ measured. Miller Units were calculated using the following calculation:

Miller Units = 1000 × OD_420nm_/(OD_600nm_ of culture samples)x(volume taken[0.02 ml])x(reaction time(min))).

At least 3 independent experiments were carried out to determine β-galactosidase activity for each strain.

### Nitrophenol assay

To quantify AstA activity, freezer stock strains were streaked onto MH agar plates supplemented with the appropriate antibiotics and grown under microaerophilic conditions for 48 h. Each strain was re-streaked onto 3 plates and then grown under microaerophilic conditions for a further 18 h. Arylsulphatase activity was measured using previously described methods[28, 64]. Briefly, each plate was scraped into 1 ml PBS using an L-shaped spreader, and then diluted with PBS (pH 7.2) to an OD_600nm_ of 1.0. Each sample was divided into 2-paired 1 ml aliquots and centrifuged at maximum speed for 5 min. For each pair, one aliquot was washed with 1 ml buffer AB1 (0.1 M Tris·Cl, pH 7.2 (Sigma)) and centrifuged at 10,000 × *g* for 5 min. The pellet was washed and centrifuged at 10,000 × *g* for 5 min, followed by resuspension in 1 ml AB1. The same process was carried out with the other aliquot of the pair using buffer AB2 (2 mM tyramine (Sigma) in 0.1 M Tris·Cl, pH 7.2). Following this, 200 µl of each paired aliquot, per sample, was added to 200 µl of freshly prepared buffer AB3 (20 mM nitrophenyl sulphate (Sigma) in 0.1 M Tris·Cl, pH 7.2) and incubated for 1 h at 37 °C. The reactions were stopped by addition of 0.2 M NaOH (Sigma) and the OD_410nm_ for each sample was measured, the samples in AB1 were used as blanks for paired samples in AB2.

A nitrophenol standard curve was prepared by performing serial 1:2 dilutions using 200 µM p-Nitrophenol (Sigma) in buffer AB1 (0.1 M Tris·Cl, pH 7.2 (Sigma)) and obtaining OD_410nm_ values for each concentration. The standard curve was then used to convert OD_410nm_ values obtained from the nitrophenol assay into µM nitrophenol released. At least 3 independent experiments were carried out to determine arylsulphatase activity for each strain.

### Hippicurase assay

To quantify HipO activity, freezer stock strains were streaked onto MH agar plates supplemented with the appropriate antibiotics and grown under microaerophilic conditions for 48 h. Each strain was re-streaked onto 3 plates and then grown under microaerophilic conditions for a further 18 h. Hippicurase activity was measured using a previously described method[32]. Each plate was scraped into 1 ml PBS using an L-shaped spreader, and then diluted with PBS to an OD_600nm_ of 1.0 and pelleted by microcentrifugation for 5 min at 10,000 × *g* at room temperature. Pellets were washed with 1 ml HIP buffer (2% w/v sodium Hippurate in 100 mM Tris–HCl, pH 8.0) and pelleted as previously described. Pellets were resuspended in 1 ml HIP buffer and incubated, along with a 1 ml cell-free control aliquot of HIP buffer, for 30 min at 37 °C. Suspensions were pelleted and 900 µl supernatant added to 500 µl ninhydrin reagent (3.5% ninhydrin in 1:1 acetone:butanol mixture) and incubated at 95 °C for 20 min. Samples were centrifuged for 1 min at 10,000 × *g* at room temperature to remove debris, and OD_570nm_ recorded using the no-cell HIP buffer-ninhydrin reagent mixture as a control. If necessary, samples were diluted in 60% ethanol before OD_570nm_ measurement. At least 3 independent experiments were carried out to determine hippicurase activity for each strain.

### Motility assays

Freezer stock strains were streaked onto MH agar plates supplemented with the appropriate antibiotics and grown ﻿under microaerophilic conditions for 48 h. Each strain was re-streaked onto 3 plates and then grown ﻿under microaerophilic conditions for a further 18 h. Motility was measured using previously described methods[25, 65] as follows.

The bacterial growth from each plate was scraped into 1 ml MH broth using an L-shaped spreader, and then diluted with MH broth to an OD_600nm_ of 0.4. Three x 1 μl aliquots of each suspension were stabbed, using a 10 μl pipette tip, into MH broth with 0.4% select agar (Sigma) to provide 3 stabs for each technical replicate, followed by 16 h growth under microaerophilic conditions. The diameter of bacterial growth was measured using a ruler. At least 3 separate biological replicates were performed for each strain tested.

### Transmission electron microscopy

*C. jejuni* strains were grown in liquid culture in MH broth until an OD_600nm_ of 0.4 was reached. 600 μl of each culture was gently pipetted into a 1.5 ml Eppendorf and 600 μl of 8% paraformaldehyde (PFA) (v/v) in PBS (pH 7.2) was added, followed by incubation at room temperature for 45 min. Following this, suspensions were washed twice with PBS, followed by centrifugation for 5 min at 4000 × *g*, and resuspended in 100 μl PBS. 20 μl suspension was adsorbed onto glow discharged 400 mesh copper grids with a carbon film attached for 30 s. The grids were rinsed twice in dH_2_O and stained for 30 s with 1% aqueous uranyl acetate (UA). Grids were then viewed using a Tecnai G2 (FEI) transmission electron microscope operated at 200 keV. Around 500–1000 bacterial cells were surveyed across each TEM grid.

### Data and statistical analysis

Statistical analysis and graphical presentation were performed using GraphPad Prism (GraphPad Software, San Diego, California USA, www.graphpad.com).

## Supplementary Information

**Additional file 1.**

**Additional file 2.**

**Additional file 3.**

## Data Availability

All data generated or analysed during this study are included in this published article [and its supplementary information files]. The datasets used and/or analysed, including TEM images, during the current study are available from the corresponding author on reasonable request.
