# Supplementary material for: Development and validation of a CRISPR interference system for gene regulation in Campylobacter jejuni
Source: BMC Microbiol. 2022 Oct 5;22:238. doi: 10.1186/s12866-022-02645-4 (PMC9533551; doi:10.1186/s12866-022-02645-4)
Supplement: Supplementary file 1 — Additional file 1. [file 12866_2022_2645_MOESM1_ESM.docx]

**Table S1. Strains used and generated within this study**

| **Strain name** | **Description/genotype** | **Source or reference** |
| --- | --- | --- |
| **C. jejuni** | | |
| M1Cam | **﻿**Derivative of M1 used in our laboratory. | (38,39) |
| 81-176 | Clinical isolate 81-176 | (40) |
| M1Cam No promoter lacZ | lacZ Kan^r^ | This study |
| 81-176 No promoter lacZ | lacZ Kan^r^ | This study |
| M1-Cam [pPorA]:lacZ | [pPorA]:lacZ Kan^r^ | This study |
| 81-176 [pPorA]:lacZ | [pPorA]:lacZ Kan^r^ | This study |
| M1Cam [pCat]:lacZ | [pCat]:lacZ Kan^r^ | This study |
| 81-176 [pCat]:lacZ | [pCat]:lacZ Kan^r^ | This study |
| M1Cam [pCas9]:lacZ | [pCas9]:lacZ Kan^r^ | This study |
| 81-176 [pCas9]:lacZ | [pCas9]:lacZ Kan^r^ | This study |
| M1Cam [pMetK]:lacZ | [pMetK]:lacZ Kan^r^ | This study |
| 81-176 [pMetK]:lacZ | [pMetk]:lacZ Kan^r^ | This study |
| M1Cam∆CRISPR | ∆CRISPR_array/CJM1cam_1464/CJM1cam_1465/CJM1_1466-1467::cat | This study |
| 81-176 AstA_47 | [PporA]AstA_47 [pCat]:dcas9 Kan^r^ | This study |
| M1Cam AstA_47 | [pPorA]AstA_47 [pCat]:dcas9 Kan^r^ | This study |
| 81-176 AstA_85 | [pPorA]AstA_85 [pCat]:dcas9 Kan^r^ | This study |
| M1Cam AstA_85 | [pPorA]AstA_85 [pCat]:dcas9 Kan^r^ | This study |
| 81-176 AstA_145 | [pPorA]AstA_145 [pCat]:dcas9 Kan^r^ | This study |
| M1Cam AstA_145 | [pPorA]AstA_145 [pCat]:dcas9 Kan^r^ | This study |
| 81-176 AstA_160 | [pPorA]AstA_160 [pCat]:dcas9 Kan^r^ | This study |
| M1Cam AstA_160 | [pPorA]AstA_160 [pCat]:dcas9 Kan^r^ | This study |
| 81-176 AstA_523 | [pPorA]AstA_523 [pCat]:dcas9 Kan^r^ | This study |
| M1Cam AstA_523 | [pPorA]AstA_523 [pCat]:dcas9 Kan^r^ | This study |
| 81-176 AstA_568 | [pPorA]AstA_568 [pCat]:dcas9 Kan^r^ | This study |
| M1Cam AstA_568 | [pPorA]AstA_568 [pCat]:dcas9 Kan^r^ | This study |
| 81-176 AstA_802 | [pPorA]AstA_802 [pCat]:dcas9 Kan^r^ | This study |
| M1Cam AstA_802 | [pPorA]AstA_802 [pCat]:dcas9 Kan^r^ | This study |
| 81-176 AstA_892 | [pPorA]AstA_892 [pCat]:dcas9 Kan^r^ | This study |
| M1Cam AstA_892 | [pPorA]AstA_892 [pCat]:dcas9 Kan^r^ | This study |
| 81-176 AstA_1091 | [pPorA]AstA_1091 [pCat]:dcas9 Kan^r^ | This study |
| M1Cam AstA_1091 | [pPorA]AstA_1091 [pCat]:dcas9 Kan^r^ | This study |
| 81-176 AstA_1212 | [pPorA]AstA_1212 [pCat]:dcas9 Kan^r^ | This study |
| M1Cam AstA_1212 | [pPorA]AstA_1212 [pCat]:dcas9 Kan^r^ | This study |
| 81-176 AstA_1368 | [pPorA]AstA_1368 [pCat]:dcas9 Kan^r^ | This study |
| M1Cam AstA_1368 | [pPorA]AstA_1368 [pCat]:dcas9 Kan^r^ | This study |
| 81-176 AstA_1774 | [pPorA]AstA_1774 [pCat]:dcas9 Kan^r^ | This study |
| M1Cam AstA_1774 | [pPorA]AstA_1774 [pCat]:dcas9 Kan^r^ | This study |
| M1Cam∆CRISPR AstA_47 | [pPorA]AstA_47 [pCat]:dcas9 Kan^r^ | This study |
| M1Cam∆CRISPR AstA_85 | [pPorA]AstA_85 [pCat]:dcas9 Kan^r^ | This study |
| M1Cam∆CRISPR AstA_145 | [pPorA]AstA_145 [pCat]:dcas9 Kan^r^ | This study |
| M1Cam∆CRISPR AstA_160 | [pPorA]AstA_160 [pCat]:dcas9 Kan^r^ | This study |
| M1Cam∆CRISPR AstA_523 | [pPorA]AstA_47 [pCat]:dcas9 Kan^r^ | This study |
| M1Cam∆CRISPR AstA_568 | [pPorA]AstA_568 [pCat]:dcas9 Kan^r^ | This study |
| M1Cam∆CRISPR AstA_802 | [pPorA]AstA_802 [pCat]:dcas9 Kan^r^ | This study |
| M1Cam∆CRISPR AstA_892 | [pPorA]AstA_892 [pCat]:dcas9 Kan^r^ | This study |
| M1Cam∆CRISPR AstA_1091 | [pPorA]AstA_1091 [pCat]:dcas9 Kan^r^ | This study |
| M1Cam∆CRISPR AstA_1212 | [pPorA]AstA_1212 [pCat]:dcas9 Kan^r^ | This study |
| M1Cam∆CRISPR AstA_1368 | [pPorA]AstA_1368 [pCat]:dcas9 Kan^r^ | This study |
| M1Cam∆CRISPR AstA_1774 | [pPorA]AstA_1774 [pCat]:dcas9 Kan^r^ | This study |
| 81-176 HipO_13 | [pPorA]HipO_13 [pCat]:dcas9 Kan^r^ | This study |
| M1Cam HipO_13 | [pPorA]HipO_13 [pCat]:dcas9 Kan^r^ | This study |
| 81-176 HipO_394 | [pPorA]HipO_394 [pCat]:dcas9 Kan^r^ | This study |
| M1Cam HipO_394 | [pPorA]HipO_394 [pCat]:dcas9 Kan^r^ | This study |
| 81-176 HipO_613 | [pPorA]HipO_613 [pCat]:dcas9 Kan^r^ | This study |
| M1Cam HipO_613 | [pPorA]HipO_613 [pCat]:dcas9 Kan^r^ | This study |
| 81-176 HipO_676 | [pPorA]HipO_676 [pCat]:dcas9 Kan^r^ | This study |
| M1Cam HipO_676 | [pPorA]HipO_676 [pCat]:dcas9 Kan^r^ | This study |
| 81-176 HipO_883 | [pPorA]HipO_883 [pCat]:dcas9 Kan^r^ | This study |
| M1Cam HipO_883 | [pPorA]HipO_883 [pCat]:dcas9 Kan^r^ | This study |
| 81-176 HipO_976 | [pPorA]HipO_976 [pCat]:dcas9 Kan^r^ | This study |
| M1Cam HipO_976 | [pPorA]HipO_976 [pCat]:dcas9 Kan^r^ | This study |
| 81-176 FlgR_246 | [pPorA]FlgR_246 [pCat]:dcas9 Kan^r^ | This study |
| M1Cam FlgR_246 | [pPorA]FlgR_246 [pCat]:dcas9 Kan^r^ | This study |
| 81-176 FlgR_290 | [pPorA]FlgR_290 [pCat]:dcas9 Kan^r^ | This study |
| M1Cam FlgR_290 | [pPorA]FlgR_290 [pCat]:dcas9 Kan^r^ | This study |
| 81-176 FlgR_559 | [pPorA]FlgR_559 [pCat]:dcas9 Kan^r^ | This study |
| M1Cam FlgR_559 | [pPorA]FlgR_559 [pCat]:dcas9 Kan^r^ | This study |
| 81-176 FlgR_647 | [pPorA]FlgR_647 [pCat]:dcas9 Kan^r^ | This study |
| M1Cam FlgR_647 | [pPorA]FlgR_647 [pCat]:dcas9 Kan^r^ | This study |
| 81-176 FlgR_913 | [pPorA]FlgR_913 [pCat]:dcas9 Kan^r^ | This study |
| M1Cam FlgR_913 | [pPorA]FlgR_913 [pCat]:dcas9 Kan^r^ | This study |
| 81-176 FlaA_761 | [pPorA]FlaA_761 [pCat]:dcas9 Kan^r^ | This study |
| M1Cam FlaA_761 | [pPorA]FlaA_761 [pCat]:dcas9 Kan^r^ | This study |
| 81-176 FlaA_1662 | [pPorA]FlaA_1662 [pCat]:dcas9 Kan^r^ | This study |
| M1Cam FlaA_1662 | [pPorA]FlaA_1662 [pCat]:dcas9 Kan^r^ | This study |
| 81-176 FlaB_761 | [pPorA]FlaB_761 [pCat]:dcas9 Kan^r^ | This study |
| M1Cam FlaB_761 | [pPorA]FlaB_761 [pCat]:dcas9 Kan^r^ | This study |
| 81-176 FlaB_1592 | [pPorA]FlaB_1592 [pCat]:dcas9 Kan^r^ | This study |
| M1Cam FlaB_1592 | [pPorA]FlaB_1592 [pCat]:dcas9 Kan^r^ | This study |
| 81-176 FlaAB_428 | [pPorA]FlaAB_428 [pCat]:dcas9 Kan^r^ | This study |
| M1Cam FlaAB_428 | [pPorA]FlaAB_428 [pCat]:dcas9 Kan^r^ | This study |
| 81-176 FlaAB_441 | [pPorA]FlaAB_441 [pCat]:dcas9 Kan^r^ | This study |
| M1Cam FlaAB_441 | [pPorA]FlaAB_441 [pCat]:dcas9 Kan^r^ | This study |
| 81-176 FlaAB_441 | [pPorA]FlaAB_441 [pCat]:dcas9 Kan^r^ | This study |
| M1Cam FlaAB_441 | [pPorA]FlaAB_441 [pCat]:dcas9 Kan^r^ | This study |
| 81-176 FlaAB_527 | [pPorA]FlaAB_527 [pCat]:dcas9 Kan^r^ | This study |
| M1Cam FlaAB_527 | [pPorA]FlaAB_527 [pCat]:dcas9 Kan^r^ | This study |
| 81-176 FlaAB_851 | [pPorA]FlaAB_851 [pCat]:dcas9 Kan^r^ | This study |
| M1Cam FlaAB_851 | [pPorA]FlaAB_851 [pCat]:dcas9 Kan^r^ | This study |
| 81-176 FlaAB_1034 | [pPorA]FlaAB_1034 [pCat]:dcas9 Kan^r^ | This study |
| M1Cam FlaAB_1034 | [pPorA]FlaAB_1034 [pCat]:dcas9 Kan^r^ | This study |
| 81-176 FlaAB_1124 | [pPorA]FlaAB_1124 [pCat]:dcas9 Kan^r^ | This study |
| M1Cam FlaAB_1124 | [pPorA]FlaAB_1124 [pCat]:dcas9 Kan^r^ | This study |
| 81-176 FlaAB_1394 | [pPorA]FlaAB_1394 [pCat]:dcas9 Kan^r^ | This study |
| M1Cam FlaAB_1394 | [pPorA]FlaAB_1394 [pCat]:dcas9 Kan^r^ | This study |
| 81-176 FlaAB_1394 | [pPorA]FlaAB_1394 [pCat]:dcas9 Kan^r^ | This study |
| M1Cam FlaAB_1394 | [pPorA]FlaAB_1394 [pCat]:dcas9 Kan^r^ | This study |
| **E. coli** | | |
| DH5α derivative | F^–^φ80lacZΔM15 Δ(lacZYA-argF)U169 recA1 endA1 hsdR17(r_K_^–^,m_K_^+^) phoA supE44 λ^–^ thi-1 gyrA96 relA1 | New England Biolabs |
| K12 derivative | F´proA+B+ lacIq Δ(lacZ)M15 zzf::Tn10(TetR)/ fhuA2 glnV Δ(lac-proAB) thi-1 Δ(hsdS-mcrB)5 | New England Biolabs |

**Table S2. Plasmids used and generated in this study**

| **Plasmid** | **Description** | **Source or reference** |
| --- | --- | --- |
| pSV009 | pUC19 backbone with MCS, kan cassette, ~400bp homologous arms to CjM1_055/0056-57 and amp cassette | (39) |
| pRC1 | pSV009 with cat promoter removed | This study |
| pRC2 | pRC1 with lacZ | This study |
| pRC3 | pRC1 with [pPorA]:lacZ | This study |
| pRC4 | pRC1 with [pCat]:lacZ | This study |
| pRC27 | pRC1 with [pCas9]:LacZ | This study |
| pRC28 | pRC1 with [pMetK]:LacZ | This study |
| pRC34 | pRC1 with AvrII site added | This study |
| pRC35 | pRC34 with [pPorA]:astA _47 guide insert | This study |
| pRC36 | pRC34 with [pPorA]:astA _85 guide insert | This study |
| pRC37 | pRC34 with [pPorA]:astA_145 guide insert | This study |
| pRC38 | pRC34 with [pPorA]:astA_160 guide insert | This study |
| pRC39 | pRC34 with [pPorA]:astA_523 guide insert | This study |
| pRC40 | pRC35 with [pCat]:dcas9 | This study |
| pRC41 | pRC36 with [pCat]:dcas9 | This study |
| pRC42 | pRC37 with [pCat]:dcas9 | This study |
| pRC43 | pRC38 with [pCat]:dcas9 | This study |
| pRC44 | pRC39 with [pCat]:dcas9 | This study |
| pRC46 | pRC35 with [pPorA]:flgR_246 guide insert | This study |
| pRC47 | pRC35 with [pPorA]:flgR_290 guide insert | This study |
| pRC49 | pRC35 with [pPorA]:flgR_647guide insert | This study |
| pRC51 | pRC35 with [pPorA]:flgR_556 guide insert | This study |
| pRC52 | pRC35 with [pPorA]:flgR_913 guide insert | This study |
| pRC56 | pRC46 with [pCat]:dcas9 | This study |
| pRC57 | pRC47 with [pCat]:dcas9 | This study |
| pRC59 | pRC49 with [pCat]:dcas9 | This study |
| pRC61 | pRC51 with [pCat]:dcas9 | This study |
| pRC62 | pRC52 with [pCat]:dcas9 | This study |
| pRC73 | pRC35 with [pPorA]:astA_568 guide insert | This study |
| pRC74 | pRC35 with [pPorA]:astA_802 guide insert | This study |
| pRC75 | pRC35 with [pPorA]:astA_892 guide insert | This study |
| pRC76 | pRC35 with [pPorA]:astA_1091 guide insert | This study |
| pRC77 | pRC35 with [pPorA]:astA_1212 guide insert | This study |
| pRC78 | pRC35 with [pPorA]:astA_1638 guide insert | This study |
| pRC79 | pRC35 with [pPorA]:astA_1774 guide insert | This study |
| pRC81 | pRC73 with [pCat]:dcas9 | This study |
| pRC82 | pRC74 with [pCat]:dcas9 | This study |
| pRC83 | pRC75 with [pCat]:dcas9 | This study |
| pRC84 | pRC76 with [pCat]:dcas9 | This study |
| pRC85 | pRC77 with [pCat]:dcas9 | This study |
| pRC86 | pRC78 with [pCat]:dcas9 | This study |
| pRC87 | pRC79 with [pCat]:dcas9 | This study |
| pRC89 | pRC35 with [pPorA]:hipO_O13 guide insert | This study |
| pRC90 | pRC35 with [pPorA]:hipO_394 guide insert | This study |
| pRC91 | pRC35 with [pPorA]:hipO_613 guide insert | This study |
| pRC92 | pRC35 with [pPorA]:hipO_676 guide insert | This study |
| pRC93 | pRC35 with [pPorA]:hipO_883 guide insert | This study |
| pRC94 | pRC35 with [pPorA]:hipO_976 guide insert | This study |
| pRC95 | pRC35 with [pPorA]:flaA_761 guide insert | This study |
| pRC96 | pRC35 with [pPorA]:flaA_1662 guide insert | This study |
| pRC97 | pRC89 with [pCat]:dcas9 | This study |
| pRC98 | pRC90 with [pCat]:dcas9 | This study |
| pRC99 | pRC91 with [pCat]:dcas9 | This study |
| pRC100 | pRC92 with [pCat]:dcas9 | This study |
| pRC101 | pRC93 with [pCat]:dcas9 | This study |
| pRC102 | pRC94 with [pCat]:dcas9 | This study |
| pRC103 | pRC95 with [pCat]:dcas9 | This study |
| pRC104 | pRC96 with [pCat]:dcas9 | This study |
| pRC105 | pRC35 with [pPorA]:flaB_761 guide insert | This study |
| pRC106 | pRC35 with [pPorA]:flaB_1592 guide insert | This study |
| pRC107 | pRC35 with [pPorA]:flaAflaB_428 guide insert | This study |
| pRC108 | pRC35 with [pPorA]:flaAflaB441 guide insert | This study |
| pRC109 | pRC35 with [pPorA]:flaAflaB527 guide insert | This study |
| pRC110 | pRC35 with [pPorA]:flaAflaB851 guide insert | This study |
| pRC111 | pRC35 with [pPorA]:flaAflaB1034 guide insert | This study |
| pRC112 | pRC35 with [pPorA]:flaAflaB1124 guide insert | This study |
| pRC113 | pRC35 with [pPorA]:flaAflaB1130 guide insert | This study |
| pRC114 | pRC35 with [pPorA]:flaAflaB1394 guide insert | This study |
| pRC115 | pRC105 with [pCat]:dcas9 | This study |
| pRC116 | pRC106 with [pCat]:dcas9 | This study |
| pRC117 | pRC107 with [pCat]:dcas9 | This study |
| pRC118 | pRC108 with [pCat]:dcas9 | This study |
| pRC119 | pRC109 with [pCat]:dcas9 | This study |
| pRC120 | pRC110 with [pCat]:dcas9 | This study |
| pRC121 | pRC111 with [pCat]:dcas9 | This study |
| pRC122 | pRC112 with [pCat]:dcas9 | This study |
| pRC123 | pRC113 with [pCat]:dcas9 | This study |
| pRC124 | pRC114 with [pCat]:dcas9 | This study |
| pRC_CRISPR_Del | pRC1 with Cat cassette between homologous arms to CRISPR_array/CJM1cam_1464/CJM1cam_1465/CJM1_1466-1467 | This study |

**Table S3. Primers used in this study**

| **Name** | **Sequence (5’-3’)** | **F/R/S** | **Description** | **Tm(°C)** |
| --- | --- | --- | --- | --- |
| ES02 | CTCGAGTTAAGTGCTTCTTAAGAAAAAACTCCAAATTTATGTGCTACAATTACGATGTTTTATTAATTTTTGACAAGGAGAATTCTCATGGTCGTTTTACAACGTCGTGACT | F | PAGE purified forward primer for addition of pPorA to lacZ sequence with XhoI site and ATG added. | 78 |
| RC18 | CTGGGATCCTTATTTTTGACACCAGACCAACTG | R | lacZ sequence reverse with BamHI site. | 72 |
| RC20 | CAGCTCGAGTATGATATAGTGGATAGATTTATGATATAATGAGTTATCAACAAATCGGAATTTACGGAGGATAAATGGTCGTTTTACAACGTCGTGAC | F | PAGE purified forward primer for addition of pCat to lacZ sequence with XhoI site and ATG added. | 77 |
| RC21 | CAGCTCGAGATGGTCGTTTTACAACGTCGTGAC | F | lacZ sequence forward with XbaI site and ATG added. | 75 |
| RC47 | AAGCCACACTAGTCGCATCA | S | Sequencing primer to check promoter:lacZ insertion into pRC1 | 66 |
| RC133 | CAGCTCGAGTATGATATAGTGGATAGATTTATGATATAATGAGTTATCAACAAATCGGAATTTACGGAGGATAAATGGATAAGAAATACTCAATAG | F | Amplification of dCas9 with cat promoter from 11168 and XhoI site | 74 |
| RC134 | CAGTCTAGATTAGTCACCTCCTAGCTG | R | Amplification of dcas9 with XbaI site | 67 |
| RC135 | CAGCCTAGGAAAGCTCGAGTACGCGTCTGTAAC | F | Amplification of pRC1 to introduce AvrII site | 76 |
| RC136 | CAGCCTAGGTCTCTCCGCTAGAAATTAA | R | Amplification of pRC1 to introduce AvrII site | 69 |
| RC137 | ATGGATAAGAAATACTCAATAGGC | F | Amplification of dcas9 | 59 |
| RC138 | TTAGTCACCTCCTAGCTGACTC | R | Amplification of dcas9 | 65 |
| RC151 | CAGACGCGTTTAAGTGCTTCTTAAG | F | Amplification of guide scaffold forward for pRC34 | 66 |
| RC152 | CAGCCTAGGAAAAAGCACCGACTC | R | Amplification of guide scaffold Reverse for pRC34 | 70 |
| RC165 | GTTTTAGAGCTAGAAATAGCAAG | R | Reverse Universal 5' phosphorylated | 58 |
| RC167 | AGTGGAGCGAAAATAGATTGGAGAATTCTCCTTGTCAAAAATT | F | Forward astA_85 5' phosphorylated | 72 |
| RC168 | TATGGATTGTCTCCTTTGACGAGAATTCTCCTTGTCAAAAATT | F | Forward astA _145 5' phosphorylated | 72 |
| RC169 | TTGACCGCTATTATTATGGAGAGAATTCTCCTTGTCAAAAATT | F | Forward astA _160 5' phosphorylated | 70 |
| RC170 | GGAAAACAATCTGGCAAAGGGAGAATTCTCCTTGTCAAAAATT | F | Forward astA _523 5' phosphorylated | 73 |
| RC171 | CAGACCGGTAGCTAAAATAGTTTCACA | F | Backbone PCR of pRC1 to eliminate kan F | 66 |
| RC172 | GAGCTCGGTACCCGGGGAT | R | Backbone PCR of pRC1 to eliminate kan R With AgeI site | 72 |
| RC174 | CAGACCGGTCTAAAACAATTCATCCAG | R | dcas9:kan Amplification with AgeI site Reverse | 67 |
| RC178 | AATCATATGCGCTAAGGCC | S | dcas9 inner primer to combine with RC47 | 62 |
| RC182 | GAAATAGCTCTTTTGGCTG | F/S | M1 deletion from genomic DNA for sequencing | 58 |
| RC183 | TACAGCTGTAATAGGACGGAT | R/S | M1 deletion from genomic DNA for sequencing | 62 |
| RC187 | TGCTATAGAAGCGGTTCGTTGAGAATTCTCCTTGTCAAAAATT | F | Forward flgR_246 5' phosphorylated | 73 |
| RC188 | AGCCTTTTGATGTTGATACTGAGAATTCTCCTTGTCAAAAATT | F | Forward flgR_290 5' phosphorylated | 71 |
| RC190 | GTTAATATCCCGCCTTTAAGGAGAATTCTCCTTGTCAAAAATT | F | Forward flgR_647 5' phosphorylated | 71 |
| RC199 | TTTGTTGCTATTAATATGGCGAGAATTCTCCTTGTCAAAAATT | F | Forward flgR_559 5'phosphorylated | 70 |
| RC200 | CCTTTAAGAGAGCGCAAAGAGAGAATTCTCCTTGTCAAAAATT | F | Forward flgR_913 5'phosphorylated | 72 |
| RC207 | TGGTGCTGTGTATTTAATTGC | R | Chromosomal insertion CjM1Cam_0055/0057 PCR Check Reverse | 61 |
| RC208 | GTAGGTGGTGCTATGGAATGGAGAATTCTCCTTGTCAAAAATT | F | Forward astA_568 5' phosphorylated | 74 |
| RC218 | AAATGGATGAAAGCGACAAAGAGAATTCTCCTTGTCAAAAATT | F | Forward astA_1091 5' phosphorylated | 71 |
| RC219 | TCAAAGTGCTGTTGTTAAAAGAGAATTCTCCTTGTCAAAAATT | F | Forward astA_1212 5' phosphorylated | 70 |
| RC220 | AGGTTATGAAAATGACAATGGAGAATTCTCCTTGTCAAAAATT | F | Forward astA_1368 5' phosphorylated | 70 |
| RC221 | TCGGTTCAAATTCAATTTAGGAGAATTCTCCTTGTCAAAAATT | F | Forward astA_1774 5' phosphorylated | 70 |
| RC222 | TTTAGTGTTGATCAAGCATTGAGAATTCTCCTTGTCAAAAATT | F | Forward astA_1825 5' phosphorylated | 70 |
| RC224 | AGTCGCATCAAGAGAAATTC | F | Chromosomal insertion CjM1Cam_0055/0057 PCR Check Forward | 60 |
| RC225 | GCTGCCTATATAGATTTTTCGAGAATTCTCCTTGTCAAAAATT | F | Forward astA_802 5' phosphorylated | 70 |
| RC226 | GATGGTAAGCATGTACGCACGAGAATTCTCCTTGTCAAAAATT | F | Forward astA_892 5' phosphorylated | 74 |
| RC229 | GAAATACTAGACTTACAAGGGAGAATTCTCCTTGTCAAAAATT | F | Forward hipO_13 5' phosphorylated | 69 |
| RC230 | GCTGAAGAGGGTTTGGGTGGGAGAATTCTCCTTGTCAAAAATT | F | Forward hipO_394 5' phosphorylated | 76 |
| RC231 | ATTTATGCTGCTTCTTTGCTGAGAATTCTCCTTGTCAAAAATT | F | Forward hipO_613 5' phosphorylated | 71 |
| RC232 | CAAAATTCAGCAGTTGTAAGGAGAATTCTCCTTGTCAAAAATT | F | Forward hipO_676 5' phosphorylated | 70 |
| RC233 | GTGACTATGAATAACGATGAGAGAATTCTCCTTGTCAAAAATT | F | Forward hipO_833 5' phosphorylated | 69 |
| RC234 | TTAATGGCAAGTGAGGATTTGAGAATTCTCCTTGTCAAAAATT | F | Forward hipO_976 5' phosphorylated | 71 |
| RC235 | TTAATGGAGTAACTATAGGAGAGAATTCTCCTTGTCAAAAATT | F | Forward flaA_761 5' phosphorylated | 68 |
| RC236 | AAGTGGTTCTTATGCAATGGGAGAATTCTCCTTGTCAAAAATT | F | Forward flaA_1662 5' phosphorylated | 72 |
| RC239 | TTAATGGAGTTGTAATTGGTGAGAATTCTCCTTGTCAAAAATT | F | Forward flaB_761 5' phosphorylated | 70 |
| RC240 | AATCAACCATAAGAGATGTAGAGAATTCTCCTTGTCAAAAATT | F | Forward flaB_1592 5' phosphorylated | 69 |
| RC241 | ATCAAGAATTCCAAATCGGCGAGAATTCTCCTTGTCAAAAATT | F | Forward flaAB_428 5' phosphorylated | 72 |
| RC242 | AATCGGCGCAAGTTCAAACCGAGAATTCTCCTTGTCAAAAATT | F | Forward flaAB_441 5' phosphorylated | 75 |
| RC243 | GTGCTCAAAGTTTTACTTCAGAGAATTCTCCTTGTCAAAAATT | F | Forward flaAB_527 5' phosphorylated | 70 |
| RC244 | CAGGAGTTCAAGCTTCTAAAGAGAATTCTCCTTGTCAAAAATT | F | Forward flaAB_851 5' phosphorylated | 71 |
| RC245 | ATCTTAGTGCTATAGGTATGGAGAATTCTCCTTGTCAAAAATT | F | Forward flaAB_1034 5' phosphorylated | 70 |
| RC246 | ATGCCGATGCTATGGGATTTGAGAATTCTCCTTGTCAAAAATT | F | Forward flaAB_1124 5' phosphorylated | 74 |
| RC247 | ATGCTATGGGATTTAATTCTGAGAATTCTCCTTGTCAAAAATT | F | Forward flaAB_1130 5' phosphorylated | 69 |
| RC248 | TTAAAACTACTGCTGCTAATGAGAATTCTCCTTGTCAAAAATT | F | Forward flaAB_1394 5' phosphorylated | 69 |
| qRC01 | CTATAGAAGCGGTTCGTTTGGGAG | F | flgR product 1 Forward | 67 |
| qRC02 | GCACGCTTAATAGCCTCGAC | R | flgR product 1 Reverse | 66 |
| qRC03 | AAACAGCTCAGCCAAGAAGC | F | flgR product 2 Forward | 66 |
| qRC04 | ACAAGCTCTTTGTACAACGGATATT | R | flgR product 2 Reverse | 64 |
| qRC07 | CATTGAATGCAAAAGCAAACTCTGATC | F | flaA product 1 Forward | 65 |
| qRC08 | AGATCTTAAGCTATCTGCTATCGCC | R | flaA product 1 Reverse | 66 |
| qRC09 | ACACCAACATCGGTGCATTAAATGCA | F | flaB product 1 Forward | 69 |
| qRC10 | GCTTGTGAACGCAAAGAATCTGC | R | flaB product 1 Reverse | 67 |
| qRC11 | CATGGCTATTATTCCAACCACGGA | F | gyrA product 1 Forward | 67 |
| qRC12 | GATCGCTCTAACTCCTACACTTCTGA | R | gyrA product 1 Reverse | 67 |
| qRC13 | AAGCAGCAGAATCGCAAATCAG | F | flaA product 2 Forward | 66 |
| qRC14 | GCTGACTAGAATTTGCTTGAGCC | R | flaA product 2 Reverse | 66 |
| qRC16 | ACAGCGTTAGCTTGACTCATAGC | R | flaB product 2 Reverse | 67 |
| qRC17 | GTAGACTTTGCAAGTGAGAGTGCG | F | flaB product 2 Forward | 68 |
