# Supplementary material for: Development and validation of a CRISPR interference system for gene regulation in Campylobacter jejuni
Source: BMC Microbiol. 2022 Oct 5;22:238. doi: 10.1186/s12866-022-02645-4 (PMC9533551; doi:10.1186/s12866-022-02645-4)
Supplement: Supplementary file 2 — Additional file 2. [file 12866_2022_2645_MOESM2_ESM.docx]

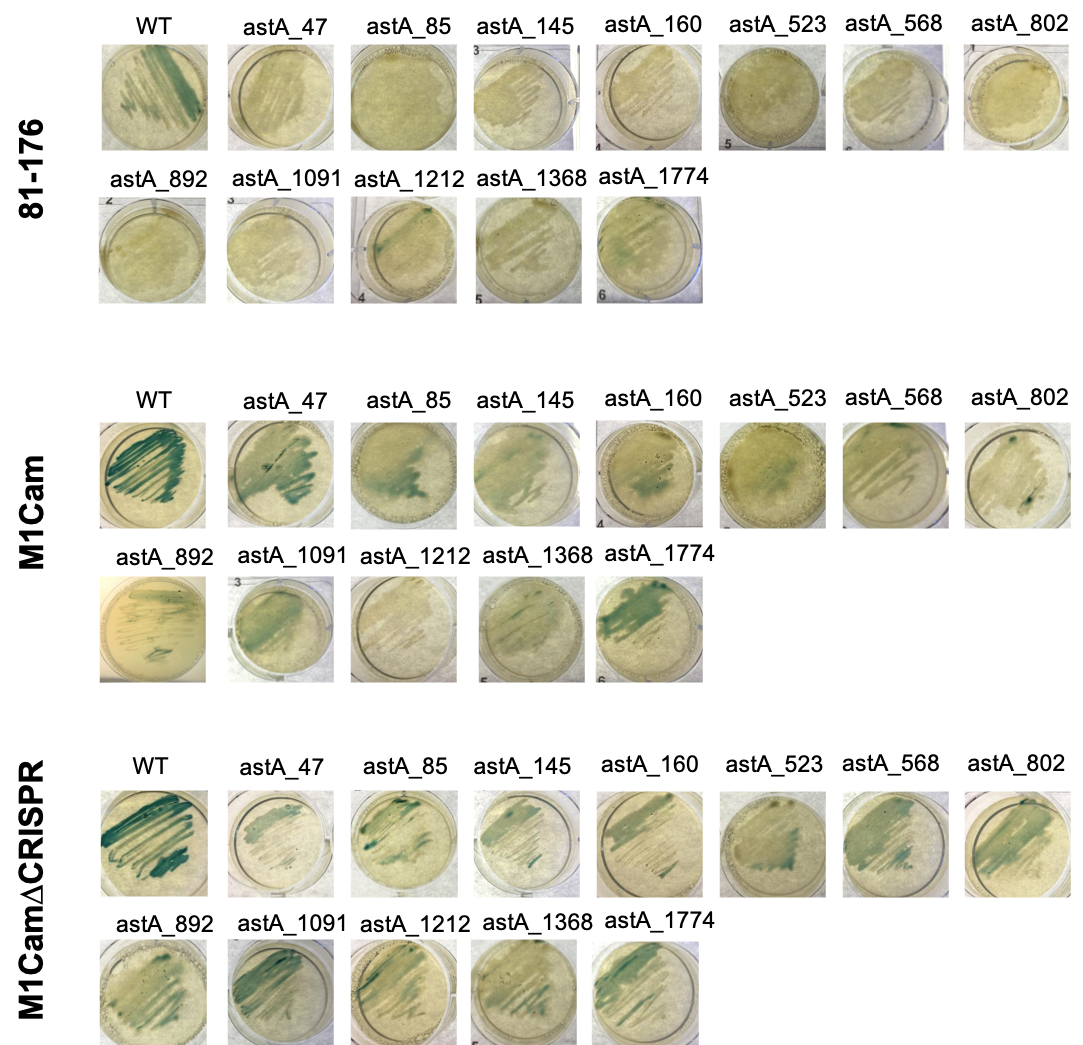


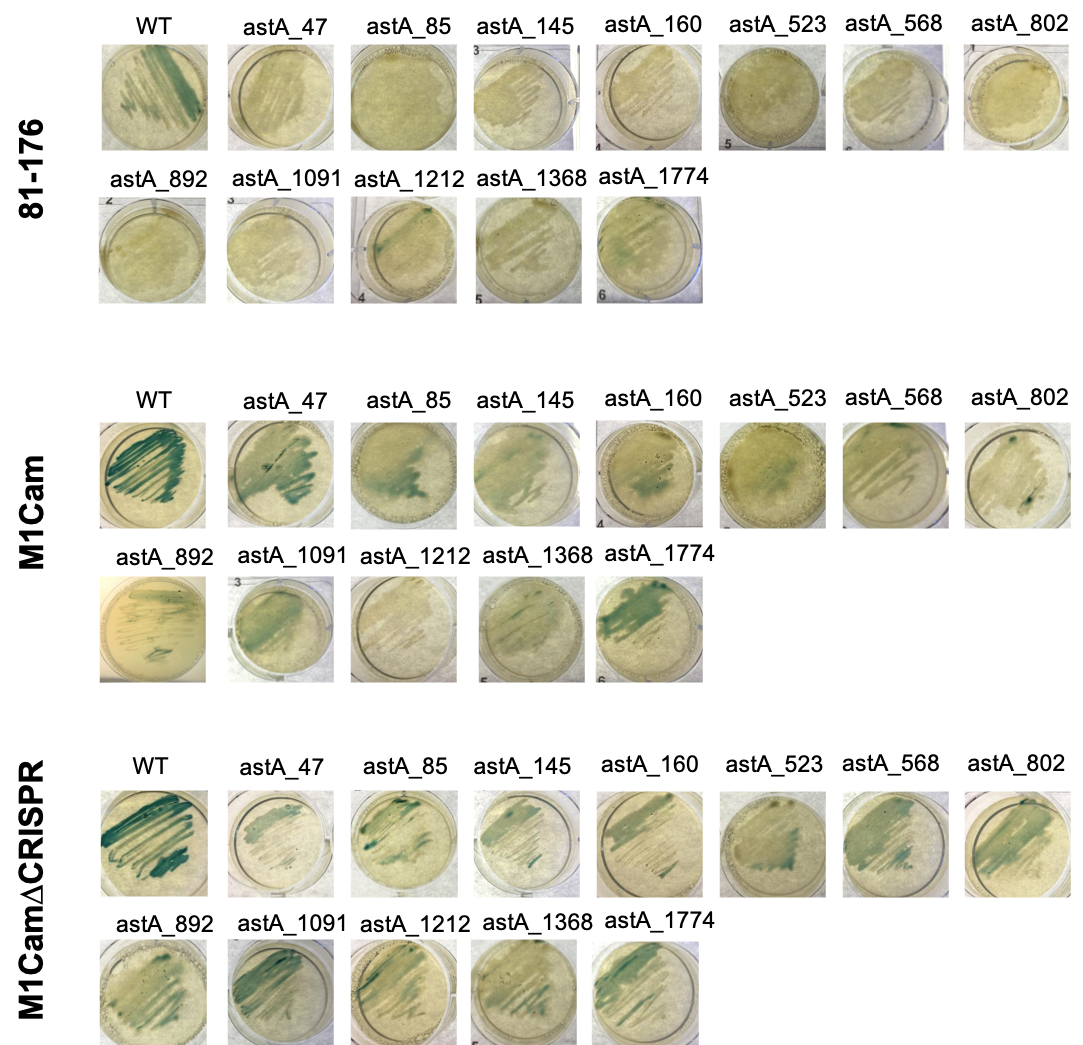


**Fig S1 Blue-white screening of 81-176, M1Cam and M1Cam∆CRISPR strains transformed to constitutively express dcas9 and guides against astA**

Strains 81-176, M1Cam and M1Cam∆CRISPR were transformed with a series of chromosomal integration CRISPRi plasmids with sgRNAs targeting 12 different locations along the astA gene. Transformants were plated on MH agar supplemented with 100µg/mL XS and incubated at 42°C under microaerophilic conditions for 48H. Transformants are each denoted as astA_N, where N is the location of the first base of the PAM site adjacent to the target region for each sgRNA.
